# Reliability and Validity of a Gamified Cognitive Assessment for Adults at High Risk of Stroke

**DOI:** 10.3390/brainsci16101125

**Published:** 2026-10-18

**Authors:** Huihong Ke, Wansi Zhong, Xiao Zhu, Rui Xue, Yiting Ge, Ying Zhou, Yi Chen, Min Lou

**Affiliations:** Department of Neurology, The Second Affiliated Hospital of Zhejiang University, School of Medicine, 88# Jiefang Road, Hangzhou 310009, China; 12318445@zju.edu.cn (H.K.); 21718233@zju.edu.cn (W.Z.); 3180101509@zju.edu.cn (X.Z.); 2326146@zju.edu.cn (R.X.); 22418367@zju.edu.cn (Y.G.); zh_ying@zju.edu.cn (Y.Z.); 2312051@zju.edu.cn (Y.C.)

**Keywords:** digital cognitive assessment, gamification, vascular cognitive impairment, test–retest reliability, convergent validity

## Abstract

**Highlights:**

**What are the main findings?**

- In older adults at high risk of stroke, a self-administered, gamified cognitive battery demonstrated stable test–retest reliability and strong agreement with conventional neuropsychological testing (canonical R = 0.86; global cognition r = 0.734).
- The battery distinguished mild cognitive impairment from normal cognition with high accuracy (AUC = 0.91) in this population, and a shortened exploratory 12-game version preserved this performance while improving feasibility.

**What are the implications of the main findings?**

- Gamified, self-administered digital assessment can serve as a scalable and user-friendly alternative to conventional cognitive testing in community and resource-limited settings.
- The tool fills a gap in digital cognitive screening for older adults at high risk of stroke, a population largely neglected by existing platforms designed for Alzheimer’s disease.

**Abstract:**

**Background**: Most existing digital cognitive assessment tools are simple adaptations of paper-and-pencil tests, lacking adequate validation in older adults and showing poor sensitivity to domain-specific deficits common in high vascular risk populations. This study aimed to evaluate the reliability, convergent validity, and user experience of a gamified Digital Cognitive Assessment Game (DCG) battery developed for adults at high risk of stroke. **Methods**: This study recruited community dwellers with high risk of stroke. Participants completed the DCG battery, a standardized neuropsychological battery, and a 2-month retest. Reliability was assessed using intraclass correlation coefficients (ICCs). Convergent validity was examined via Pearson correlations and canonical correlation analysis (CCA). User experience was evaluated using Likert-scale questionnaires. **Results**: A total of 86 participants (mean age 67 years; 60.2% female; mean education 11.37 years) completed baseline assessments. Furthermore, 12/18 DCG tasks showed acceptable-to-high test–retest reliability (ICC range: 0.795–0.883), while the remaining 6 tasks showed marginally acceptable reliability (ICC range: 0.615–0.737). Convergent validity revealed correlations in five domains (r = 0.373–0.582, *p* < 0.05), with the exception of visuospatial function (r = 0.260, *p* = 0.063), and a strong correlation for global cognition (r = 0.734, *p* < 0.001). CCA demonstrated a strong overall association between DCG and conventional batteries (R = 0.86, *p* < 0.001). The DCG showed good ability for discriminating battery-defined mild cognitive impairment (AUC = 0.91, 95% CI: 0.85–0.97). Participants reported good usability and high acceptance (80% reported having no difficulties completing DCG). **Conclusions**: The DCG battery demonstrates satisfactory reliability, validity, and user acceptability in adults at high risk of stroke. As a self-administered tool, it represents a promising approach for cognitive screening, particularly in resource-limited settings.

## 1. Introduction

An estimated 50 million people worldwide are affected by dementia, and this number is projected to reach 152 million by 2050 [1]. Among older adults, the prevalence is estimated at 5–7% [2], imposing substantial burdens on individuals, families, and healthcare systems [3]. The 2024 Lancet Commission report estimated that the risk of dementia doubles with every five-year increase in age after 60 years [4]. Given the limited effectiveness of treatments at the clinical stage [5], early identification of mild cognitive impairment (MCI) is critical for timely intervention and dementia prevention. This is particularly important in populations with high vascular risk, who are more susceptible to vascular cognitive impairment and related domain-specific deficits.

A key challenge in early detection is identifying subtle, domain-specific cognitive deficits [6]. Widely used screening tools, such as the Mini-Mental State Examination (MMSE) [7] and Montreal Cognitive Assessment (MoCA) [8], rely on global scores and single cut-offs, which may limit their sensitivity to impairments across specific cognitive domains. In contrast, comprehensive neuropsychological batteries, such as the National Institute of Neurological Disorders and Stroke and Canadian Stroke Network (NINDS-CSN) harmonization standards, provide domain-level assessment but are time-consuming, require trained personnel, and are difficult to implement in large-scale or community settings [9,10]. Therefore, there is a need for cognitive assessment tools that are both sensitive to domain-specific deficits and practical for scalable use.

Digital games are increasingly explored as scalable platforms for cognitive assessment, combining low cost, non-invasiveness, and engaging user experiences that could support both testing and cognitive training. However, several challenges remain in directly applying existing digital games to older adults in China. First, digital game apps designed for validated cognitive testing remain scarce [11]. Popular applications, such as NeuroNation, primarily focus on cognitive training functions rather than providing standardized assessments [11,12]. Second, few cognitive tools outside clinical research settings are validated for independent, unsupervised use, and platforms such as CogState typically require controlled conditions and expert interpretation, limiting their accessibility outside clinical environments [13,14]. Third, a substantial proportion of commercially available cognitive games are developed for younger users, which may reduce their usability and engagement in older adults, a highly prioritized demographic for cognitive assessment with an elevated risk of dementia [15].

To address these gaps, we developed an application called Digital Cognitive Assessment Game (DCG), a tablet-based, self-administered cognitive assessment battery integrating gamified paradigms with domain-specific tasks, tailored for older Chinese adults. This game battery was designed on the basis of previously developed cognitive training games and relevant published literature, and revisions were made according to users’ feedback, ultimately leading to the development of a digital cognitive training tool comprising 18 games, referred to as DCG, which was used in this study.

The present study aimed to evaluate whether the DCG can serve as a reliable and valid alternative to conventional neuropsychological assessments in elderly individuals at high risk of stroke.

## 2. Materials and Methods

### 2.1. Study Design

This study employed a prospective design. Participants underwent a baseline assessment (T1) and a two-month follow-up assessment (T2). Participants were recruited between January 2025 and January 2026 from the CIRCLE registry (ClinicalTrials.gov ID: NCT03542734), a single-center prospective observational study of adults with and without cerebral small vessel disease who do not have known dementia [16]. CIRCLE enrollment required participants to be aged over 40 years and to have no known dementia or stroke, although patients with acute lacunar stroke at least 6 months after onset were eligible [16]. Individuals were excluded if they had any contraindication to magnetic resonance imaging, a history of severe head injury with loss of consciousness or prior intracranial surgery, or cancer [16]. For the present analysis, we selected older adults at high risk of stroke, defined as age ≥ 55 years with at least three vascular risk factors, including hypertension, dyslipidemia, diabetes, atrial fibrillation or valvular heart disease, smoking, overweight or obesity, physical inactivity, family history of stroke, or prior transient ischemic attack.

### 2.2. Ethics

This study has been approved by the Human Ethics Committee of the Second Affiliated Hospital of Zhejiang University (Approval Code: YAN 2017-010, Approval Date: 14 April 2017). This study was conducted as part of the CIRCLE registry (NCT03542734). Written informed consent was obtained from all participants.

### 2.3. Procedure

Eligible participants from the CIRCLE study were invited to complete the DCG. Consenting participants underwent both digital cognitive assessments and traditional paper-based cognitive evaluations. These assessments were administered in a non-fixed sequence, with an interval of at least 30 min between them to minimize fatigue and potential carryover effects. Given the observational design, test order was not randomized; however, this approach reflects real-world clinical practice. After the baseline assessment, 43 of the 86 participants were randomly selected and invited to complete the DCG again after 2 months. Random selection was performed using a computer-generated random number sequence (R version 4.3.2) with simple random sampling without replacement. All 43 invited participants completed the retest.

### 2.4. Digital Cognitive Assessment Game Measures

The DCG battery was developed through a narrative, non-systematic review of the literature. The review focused on three sources: (1) published studies on digital cognitive assessment and gamified cognitive training in older adults; (2) the NINDS-CSN vascular cognitive impairment harmonization standards and related literature on domain-specific deficits in vascular cognitive impairment; and (3) previously developed cognitive training games from our group. The objective was to identify cognitive domains that are both commonly affected in older adults at high vascular risk and feasible to assess via tablet-based, self-administered gamified tasks.

The DCG was deployed on tablets, primarily considering the operability and user-friendliness for the elderly; only the Windows operating system was currently compatible. The DCG battery consists of 18 gamified tasks covering six cognitive domains (Supplementary Table S1). Detailed task procedures, scoring rules, and stimuli are provided in the Supplementary Materials. Briefly, each task requires participants to complete specific cognitive challenges, with performance quantified using accuracy, reaction time, or composite scores depending on task characteristics (Supplementary Table S1).

For digital cognitive game assessments, a researcher explained the general procedure, and each game began with a looping instructional video before testing. Participants clicked a confirm button to start only after they felt they understood the rules. During testing, participants completed the tasks independently, and the researcher’s role was limited to monitoring whether participants watched the instructional video and operated the tablet themselves. No hints or assistance were provided during task performance. Before the formal assessment, 1–2 practice games were used to help participants understand the required operations. The testing sequence of the 18 DCGs was fixed and consistent across all sessions. Although the assessment was designed to take approximately 20 min, the observed median completion time was 28 min at baseline and 26 min at retest in this sample of older adults. Scores, accuracy rates, and time spent on each game are systematically recorded. The accuracy rate or reaction time of each DCG item was calculated for cognitive score.

### 2.5. Paper-And-Pencil Neuropsychological Assessment Measures

A modified NINDS-CSN vascular cognitive impairment battery was administered by trained researchers using the 60 min protocol [9,10]. This complete battery assessed six cognitive domains: attention, executive function, language, visuomotor speed, visuospatial function and memory. Global cognition was computed from these six domains.

The complete battery is designed and validated for vascular cognitive impairment and assesses the following six cognitive domains:

(1) Attention: Digit Span Forward and Backward.
(2) Executive function: Verbal Fluency, Color Trail Test A and B.
(3) Language: 15-item Modified Boston Naming Test.
(4) Visuomotor speed: Symbol Digit Modalities Test.
(5) Visuospatial function: Rey Complex Figure Test Copy.
(6) Memory: Rey Complex Figure Test immediate/delayed recall and recognition, Hopkins Verbal Learning Test immediate/delayed recall and recognition.

### 2.6. Cognitive Status Diagnosis

Cognitive status (MCI vs. No Cognitive Impairment (NCI)) was determined based on the actuarial neuropsychological criteria proposed by Jak and Bondi [17]. Participants were classified as having MCI if they met either of the following criteria based on their performance on the NINDS-CSN battery: (1) impairment (z-score < −1.0) on both measures within at least one cognitive domain; or (2) impairment on at least one measure in each of three or more distinct cognitive domains [17]. For domains with only one available measure (language, visuomotor speed, visuospatial function), the first criterion could not be applied. Impairment in these domains was therefore defined by a single measure with z < −1.0 and contributed only to the second criterion (impairment across three or more domains).

### 2.7. Statistical Analysis

Test–retest reliability of the DCG tasks was assessed using Pearson correlations and two-way mixed-effects intraclass correlation coefficients (ICCs) for absolute agreement based on single measures, with 95% confidence intervals (CIs) computed from T1 and T2 scores. Paired-samples *t*-tests (two-tailed) were used to examine whether performance changed significantly between the two time points. Effect sizes were estimated using Cohen’s d for dependent samples, with values of 0.20, 0.50, and 0.80 interpreted as small, medium, and large, respectively.

Convergent validity was first examined using Pearson correlations between individual DCG tasks and paper-and-pencil measures. Canonical correlation analysis (CCA) was then used to assess the association between the two sets of variables (DCG and paper-and-pencil). CCA identifies linear combinations of variables that maximize the correlation between the two sets, and it allows the variables contributing most strongly to this association to be identified. CCA included 18 DCG and 14 paper-and-pencil measures (32 variables in total) in 86 participants. Given the high variable-to-sample ratio (32/86), the first canonical correlation should be interpreted as an upper-bound estimate. Significance of canonical roots was tested using Bartlett’s sequential test based on Wilks’ lambda. Bootstrap resampling with 1000 replicates was performed to assess the stability of the canonical correlation analysis. Associations between demographic variables (age, sex, and years of education) and cognitive measures were also examined. Discriminant validity was assessed using receiver operating characteristic (ROC) analysis, and the area under the curve (AUC) was calculated to evaluate the ability of the DCG to distinguish MCI from NCI. The optimal cut-off value was identified using the Youden index, defined as J = max (sensitivity + specificity − 1). Discriminant indices, including sensitivity, specificity, positive and negative predictive value (PPV and NPV), were calculated using the optimal cut-off value. Bootstrap with 1000 repeats and repeated 5-fold cross-validation was performed for ROC.

Before conducting analyses, data were screened for normality and linearity. For the DCG, game scores at each time point were standardized into z-scores using the baseline (T1) mean and standard deviation (SD). Reaction time was log-transformed due to highly skewed distribution and reversed so that higher scores reflected better performance. Domain z-scores were obtained from averaging the game/item z-scores and then standardized. Global composite scores were computed by averaging the standardized scores from 6 domain z-scores and then standardized. For the reduced 12-item battery, the calculation of domain score and global score was kept the same as the 18-item battery. For paper-and-pencil tests, item-z-score, domain-z-score and global cognition scores were obtained using normative means and standard deviations of a large sample consisting of 568 non-demented participants from the CIRCLE registry recruited before this study, not included in the present study. For multiple comparisons, the Benjamini–Hochberg (BH) correction was applied separately within each predefined family of tests. Family 1 consisted of paired-samples *t*-tests; the 18 paired-samples *t*-tests comparing T1 and T2 DCG scores were treated as one family. Family 2 consisted of task-level convergent validity analyses; Pearson correlations between individual DCG tasks and paper-and-pencil measures were corrected within each cognitive domain. Family 3 consisted of domain-level and global convergent validity analyses; the 7 domain/global comparisons were treated as one family. Family 4 consisted of demographic associations; associations between demographic variables and cognitive measures were corrected separately for DCG and NINDS-CSN. Significance level was set at *p* < 0.05. R version 4.3.2 was used for all analyses.

## 3. Results

### 3.1. Participants

The participant flowchart is shown in Figure S1. Of the 93 participants from the CIRCLE Registry who were eligible and invited to participate, 86 (92.5%) completed both the DCG and the paper-and-pencil NINDS-CSN assessments, while 7 (7.5%) did not complete the DCG (5 were unable to operate the tablet independently and 2 had visual difficulties). Among those who completed the assessments, 43 participants were randomly selected and invited for a second assessment for test–retest analysis. Baseline characteristics are shown in Supplementary Table S2. The mean age of the participants was 67 years, and 60.2% were female. The mean years of education were 11.37 (±3.56). The mean (Chinese Mini-Mental Status, CMMS) and MoCA scores were 27.43 and 23.86, respectively [18]. There was no significant difference between the retest group and the non-retest group.

### 3.2. DCG Test Performance, Practice Effects and Test–Retest Reliability

Median interval between baseline DCG and paper-and-pencil assessment was 45 (IQR 35–62) min, and the median completion times of DCG at T1 and T2 were 28 and 26 min. Median interval between DCG assessments at T1 and T2 was 58 days (IQR 50–65).

Test performance and results of the paired *t*-test for DCG are reported in Table 1. The raw paired-samples *t*-tests showed significant changes for these two tasks (Rapid Flash Memory and Visual Pattern Memory) with small-to-medium practice effects (Cohen’s d: 0.364 and 0.375 respectively), but neither remained significant after BH correction across the 18 paired-samples *t*-tests.

Test–retest reliability between T1 and T2 is presented in Table 1. Most tests displayed stable reliability across the two tests. Across 18 games, 12 showed acceptable-to-high test–retest reliability (ICCs 0.795 to 0.883). The remaining 6 items had reliability values in the marginally reliable range (ICCs 0.615 to 0.737). The global composite ICC was 0.901 (95% CI: 0.826–0.945), and domain-level ICCs ranged from 0.793 (visuospatial function) to 0.904 (memory) (Supplementary Table S3).

### 3.3. Validity

#### 3.3.1. Convergent Validity: Pearson Correlations Among DCG and Paper-And-Pencil Tests

Pearson correlation was performed to show convergent validity between DCG and paper-and-pencil tests in Table 2 and Supplementary Table S4. For most DCG items, except for Mental Arithmetic Speed and Angles, small to moderate correlations (r: from 0.226 to 0.528, *p* < 0.05) were observed for games and paper-and-pencil tests measuring the same cognitive domains. Meanwhile, domain-level correlations between DCG and paper-and-pencil tests were similar or higher (r: from 0.373 to 0.582, *p* < 0.05), with only visuospatial function presenting a borderline correlation (r = 0.260, *p* = 0.063). Of note, global cognition was strongly correlated between the two tests (r = 0.734, *p* < 0.001).

#### 3.3.2. Canonical Correlation Analyses

CCA was conducted using 14 paper-and-pencil measures and 18 DCG measures. The first pair of canonical variates was significantly correlated (R = 0.857, 95% bootstrap CI: 0.886–0.971, *p* < 0.001; canonical R^2^ = 0.73). This finding indicates a strong association between the DCG and paper-and-pencil batteries.

Table 3 presents the structure correlations between each DCG and paper-and-pencil variable and its corresponding canonical variate, ordered from highest to lowest. For DCG, moderate-sized correlations (r: 0.45–0.68) were observed for most games of the 14 measures, especially for Rapid Flash Memory, Parity Discrimination and Rapid Comparison (all r > 0.60). For paper-and-pencil measures, strong correlations (r: 0.68–0.78) were observed for Color Trail Test B, Symbol Digit Modalities Test, Digit Span Backward and Hopkins Verbal Learning Test Immediate Recall, and moderate correlations (r: 0.42–0.65) were observed for the remaining 10 measures. Overall, the strongest contributors across both batteries were tests of visuomotor speed, executive function and memory, while language measures and visuospatial function were weaker.

#### 3.3.3. ROC for Discriminating Among Participants with MCI

**Figure 1:**
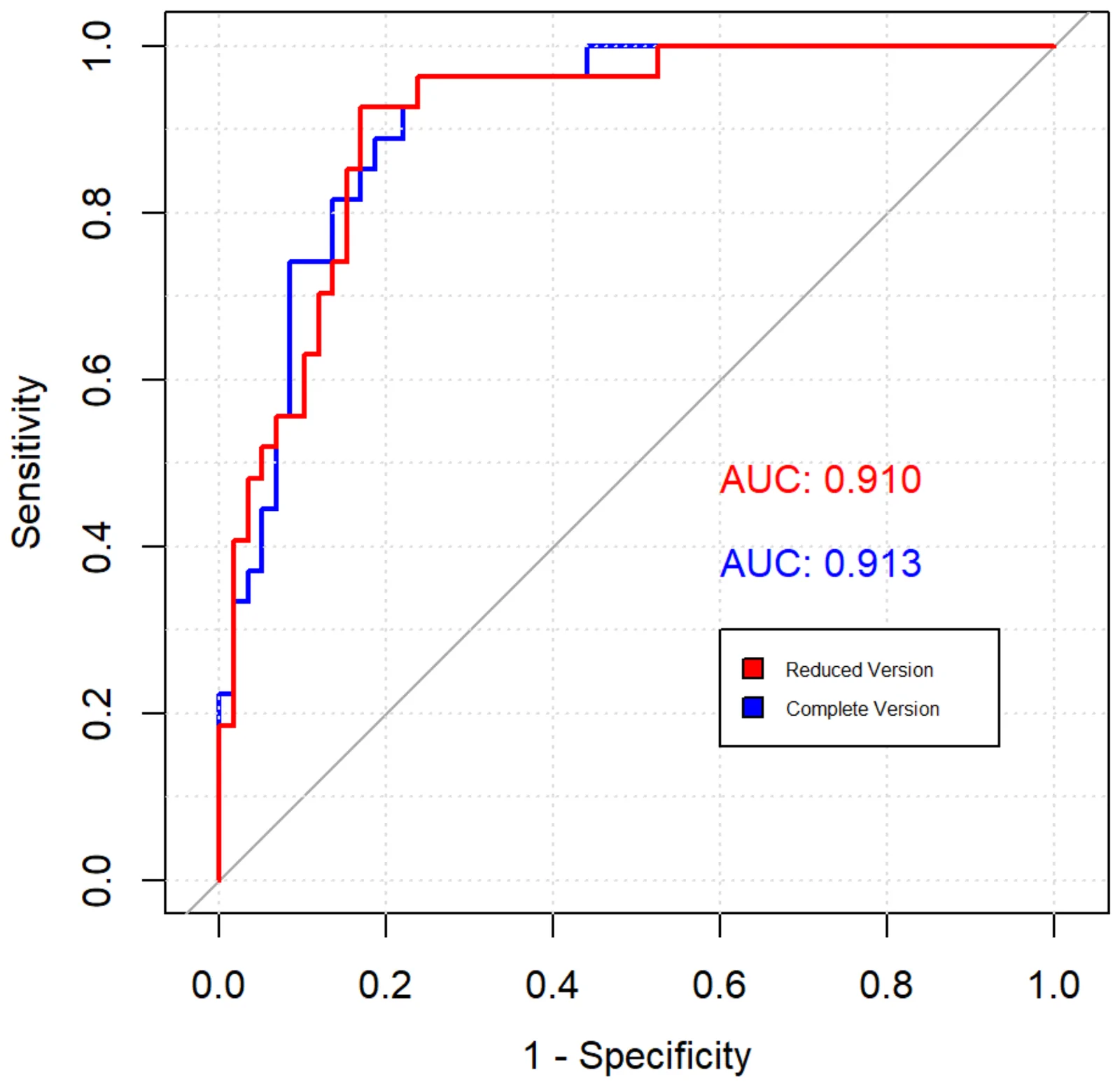
Receiver operating characteristic (ROC) curves of the DCG for discriminating among participants with MCI. The gray diagonal line represents the reference line of random chance (AUC = 0.5). The red and blue lines indicate the reduced and complete versions, respectively.

Among the 86 participants, 27 (31.4%) were classified as MCI and 59 (68.6%) as NCI based on the Jak–Bondi actuarial neuropsychological criteria. The ROC curves of DCG for distinguishing battery-defined MCI from NCI are shown in Figure 1. The AUC was 0.91 (95% CI: 0.85–0.97), and the optimal cut-off value for the global z-score was determined to be 0.22, based on the maximization of the Youden index. At this cut-off, the confusion matrix was as follows: 26 participants were true positives and 45 were true negatives (Supplementary Table S5), and the DCG demonstrated a sensitivity of 0.96 (95% CI: 0.82–0.99), a specificity of 0.76 (95% CI: 0.64–0.85), a PPV of 0.65 (95% CI: 0.50–0.78), and an NPV of 0.98 (95% CI: 0.89–1.00) (Supplementary Table S6).

### 3.4. User Experience

**Figure 2:**
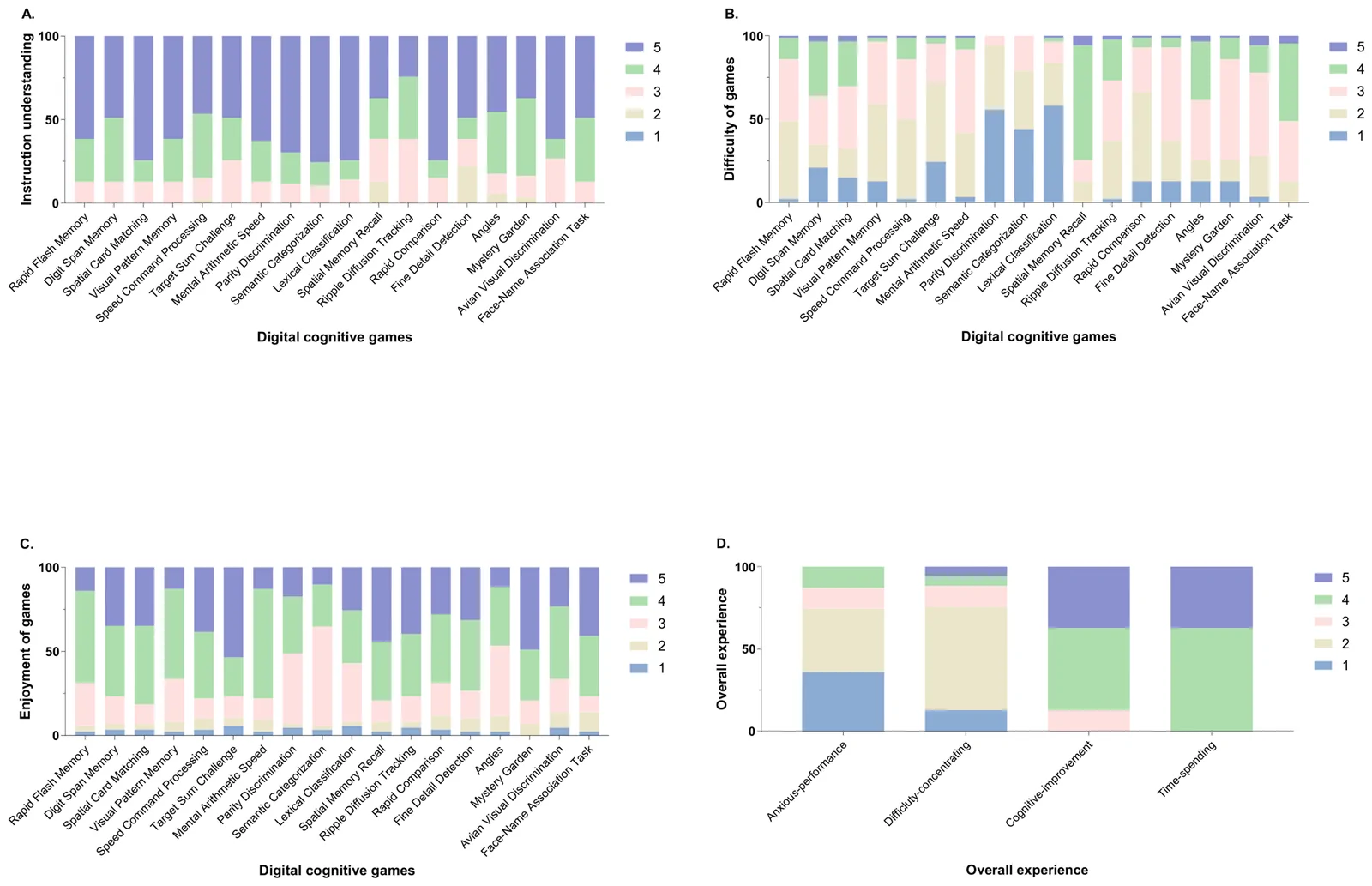
Response frequencies to three task-level user-experience items and four items assessing overall experience with the DCG. All items were rated at T1 on a 5-point Likert scale (1 = strongly disagree, 2 = disagree, 3 = neither agree nor disagree, 4 = agree, 5 = strongly agree). (**A**) The instructions were easy to understand. (**B**) The game was difficult. (**C**) I enjoyed the game. (**D**) Overall Experience with the DCG: (i) I felt anxious about my performance, (ii) I found it difficult to stay focused, (iii) I believe this gamified training can help improve cognitive function, and (iv) I am willing to spend time using this game every day for cognitive training.

Item-level responses are shown in Figure 2. Regarding ease of understanding of instructions, Semantic Categorization had the highest ratings, with nearly 90% of participants selecting “agree” or “strongly agree”, followed by Parity Discrimination (88%). Very few participants disagreed or strongly disagreed that instructions were easy to understand, with the exception of Fine Detail Detection (22%), Spatial Memory Recall (13%) and Angles (6%). The tasks most frequently rated as difficult were Spatial Memory Recall (74% agree or strongly agree) and Face-Name Association Task (51%), followed by Angles (38%) and Digit Span Memory (36%). Parity Discrimination was rated the least difficult, with 94% of participants selecting “disagree” or “strongly disagree”. Enjoyment was high for most tasks, with 14 of 18 games receiving “agree” or “strongly agree” ratings from at least 65% of participants. Lower enjoyment was reported for Semantic Categorization (35%) and Angles (47%).

Regarding overall experience with the DCG, 80% reported no difficulties using the tablets to complete the assessment; 25% had difficulty concentrating on the games and 12% reported feeling anxious about their performance (Figure 2). More than 85% of participants expressed willingness to spend some time on DCG. To examine whether user-experience ratings were associated with task performance, Spearman correlations with Benjamini-Hochberg correction were performed. No significant associations were observed after correction (all adjusted *p* > 0.05; Supplementary Table S9).

### 3.5. Associations with Demographic Variables

Few significant associations were found between demographic variables and baseline cognitive performance. However, years of education was associated with several DCG and NINDS-CSN measures. For DCG, the years of education were associated with Face-Name Association Task (r = 0.364, *p* = 0.01), Parity Discrimination (r = 0.364, *p* = 0.01), Semantic Categorization (r = 0.375, *p* < 0.01) and Lexical Classification (r = 0.401, *p* < 0.01, Supplementary Table S7). For NINDS-CSN, the years of education were associated with Verbal Fluency, Modified Boston Naming Test, Symbol Digit Modalities Test, Rey Complex Figure Test Delayed Recall and Hopkins Verbal Learning Test Immediate Recall (all *p* < 0.05, Supplementary Table S7).

### 3.6. Post Hoc Exploratory Analysis of a Reduced Set of DCG Measures

To derive a brief version of the DCG, six tasks showing practice effects or weak convergent validity were removed and a post hoc exploratory analysis for the 12-item version was performed. The excluded games and their respective rationales are listed in Table S8. Specifically, Rapid Flash Memory was excluded because of low test–retest reliability (ICC = 0.619, 95% CI: 0.339–0.851) and a moderate raw practice effect (Cohen’s d = 0.364). Visual Pattern Memory was excluded because of a moderate practice effect (Cohen’s d = 0.375). Mental Arithmetic Speed, Semantic Categorization, and Angles were removed for weak associations with paper-and-pencil measures. Spatial Memory Recall was removed due to low understandability of task instructions and relatively low validity.

Convergent validity for the 12-item DCG remained as high or slightly better for the reduced battery on six cognitive domains and global cognition (Table 2), while an obvious elevation was observed on visuospatial function (r: from 0.260 to 0.320, *p* = 0.023) and Pearson correlation of global cognition was also improved (r: from 0.734 to 0.751, *p* < 0.001). The mean cross-validated AUC was 0.91 (95% CI: 0.89–0.94) for the 12-item battery, and no significant difference in AUC was found between the 18-item and 12-item DCG (Figure 1).

## 4. Discussion

This study demonstrates that the DCG battery provides reliable, valid, and user-friendly cognitive assessment for older adults at high risk of stroke. Moreover, the 12-item DCG was developed as a brief version with comparable reliability and validity in the exploratory analysis. The findings support its potential as an efficient and scalable alternative to conventional neuropsychological testing, particularly for community-based and resource-limited settings.

### 4.1. Reliability

Overall, the DCG battery demonstrated acceptable-to-high test–retest reliability across most tasks, indicating stable performance over time. The relatively long retest interval (approximately two months) may have reduced immediate recall effects but also introduced potential variability due to true cognitive changes or external factors. Despite this, reliability remained robust for the majority of tasks, supporting the temporal stability of the assessment. However, six tasks showed relatively weak reliability, which may be attributed to increased familiarity with task rules, natural fluctuations in cognitive state, as well as the greater measurement error associated with these tasks’ limited number of trials. Notably, several low-difficulty tasks, such as Semantic Categorization and Parity Discrimination, showed scores that were generally close to the maximum, suggesting a potential ceiling effect. The reliability estimates for these tasks may therefore have been affected and should be interpreted with caution.

### 4.2. Practice Effect

Small-to-medium practice effects were observed in selected tasks, particularly Rapid Flash Memory and Visual Pattern Memory. These findings suggest that repeated exposure may facilitate familiarity with task structure and response strategies, leading to improved performance. The rules of these two games are relatively similar, primarily requiring participants to capture the spatial locations of briefly presented patterns. To attenuate the potential influence of participants memorizing the sequence or spatial positions of the patterns, both tasks were designed using randomized algorithms, ensuring that the order and location of stimuli were not fixed across trials. Nevertheless, both games still showed score improvement after two months, which may be related to participants’ improved familiarity with task rules, tablet interface operation, and response strategies. Observed improvements may partly reflect explicit strategy learning—such as developing a conscious approach to help remember and respond [19]. These practice effects could complicate the interpretation of scores in longitudinal assessments. Alternate forms with matched difficulty could be developed for tasks showing practice effects, so that repeated testing does not rely on the same stimulus set. In addition, a longer pre-assessment practice phase or a dual-baseline design could be used to reduce first-session learning effects before the true baseline is established.

### 4.3. Validity

In the present study, the global cognition of the DCG showed strong convergent validity with traditional paper-and-pencil neuropsychological assessments and demonstrated a sensitivity of 96% and a specificity of 76% in distinguishing battery-defined MCI from NCI (AUC = 0.91). Several digital platforms, such as CANTAB, Cognigram, and Neurotrack, have been validated primarily for Alzheimer’s disease-related MCI rather than VCI and few validated tools have been developed specifically for VCI [20,21,22]. ReCOGnAIze, a tablet-based tool designed to detect VCI and differentiate it from non-vascular cognitive impairment, achieved an AUC of 0.85 [23]. However, ReCOGnAIze was validated in a multi-ethnic Southeast Asian cohort with MRI-defined white matter hyperintensity burden, whereas the DCG was validated in a Chinese cohort at high vascular risk [23]. Direct comparison between the two tools is therefore limited by differences in study populations, reference standards, task composition, and scoring approaches.

CCA showed a strong association between the two assessment modalities, with the first pair of canonical variates sharing 73% of their variance. Notably, visuomotor speed, executive function, and memory emerged as the primary contributors across both batteries. This pattern is consistent with the cognitive profile commonly observed in populations at high risk of vascular cognitive impairment, in which processing speed and executive function deficits are prominent [24]. These findings support the domain sensitivity of the DCG battery for populations at high risk of VCI.

The visuospatial domain showed the weakest convergent validity (complete battery: r = 0.260, *p* = 0.063; reduced battery: r = 0.320, *p* = 0.023). This may reflect the limited number of visuospatial tasks in both batteries and differences in task demands, since RCFT Copy primarily assesses constructional ability, whereas the DCG tasks assess angle estimation and spatial arrangement.

### 4.4. Feasibility and User Experience

Although the observed administration time was longer than the intended 20 min, 26–28 min remains acceptable for a self-administered, domain-specific assessment in community-dwelling older adults, particularly given that it replaces a 60 min paper-and-pencil battery. The longer-than-expected duration may reflect the additional time required for older participants to watch the instructional videos, understand instructions, and become familiar with the tablet interface.

The battery was usable by most participants in this sample, but a small proportion of older adults with visual or operational difficulties may require assistance or may be unable to complete it independently. User-experience, reliability, and validity data were therefore available only for completers, which may have introduced selection bias and overestimated usability and accessibility. In addition, all DCG assessments were conducted in a supervised research setting, with a researcher present to set up the tablet and confirm that participants watched the instructional video, although no hints or assistance were provided during task performance. Independent community or home use requires separate validation.

In addition to its psychometric properties, the DCG demonstrated good usability and high acceptance among older participants. Most individuals reported that the tasks were understandable and engaging, and a majority expressed willingness to use the platform. This highlights the advantage of assessment with game-like experiences in improving user engagement, which is particularly important in elderly populations who may otherwise experience fatigue or reduced motivation during conventional testing [12,25]. Nevertheless, 12% of participants reported experiencing performance anxiety during the testing process, which is lower than the figure in traditional cognitive testing environments (nearly 30%) [26].

### 4.5. Reduced Battery

The post hoc development of a reduced 12-item DCG battery further improved feasibility while maintaining comparable validity in the exploratory analysis. Although repeated cross-validation and bootstrap-based optimism correction suggested stable discriminative performance, these findings should be interpreted with caution, as the 12-item battery was derived and evaluated in the same sample, and independent external validation is required before it can be recommended for clinical or community use.

### 4.6. Strengths and Limitations

Compared with existing digital cognitive tools, this study extends the literature by providing a self-administered, gamified assessment with demonstrated reliability, validity, and user acceptability in an elderly population at high risk of vascular cognitive impairment. Also, our population with a mean age of 67 years and education of 11 years may be representative due to higher cognitive impairment prevalence in the elderly and less cognitive reserve, which may be generalized to older Chinese adults who receive less education [27,28]. It has been reported that only 14% of the older adult population in China have attained at least a junior high school education level [29]. Although certain cognitive games developed abroad, such as Mind Gamepack, have demonstrated high adherence and efficacy, their validation and applicability were heavily dependent on participants’ higher educational attainment (average educational years of 17), which may render them less suitable for the Chinese population [30]. We also made cultural adaptations to certain games to enhance their suitability for the Chinese population, particularly for tasks involving language, naming, and memory for socially familiar material. In the Face-Name Association Task, we used commonly encountered traditional Chinese characters and Chinese facial features as stimuli, rather than Western faces, to reduce cultural bias in encoding and recognition. In the language tasks, we selected words from everyday Chinese vocabulary and excluded low-frequency or culturally unfamiliar items. Task instructions were simplified and delivered through short, looping instructional videos with large visual cues, which was intended to reduce the impact of low education and low digital literacy, both of which are common in older Chinese adults. The tablet interface used large touch targets and minimal text to accommodate older users with limited prior tablet experience. The integration of domain-specific tasks with a user-friendly interface with rich visual content and sound effects represents a meaningful advancement over simple digital adaptations of traditional tests. In addition, unlike the MoCA and MMSE, the NINDS-CSN battery and the DCG used in this study provide a more detailed assessment of specific cognitive domains, making them more suitable for populations at high risk of vascular impairment.

This study has several limitations. First, it was conducted as a single-center study with a relatively small sample size that was determined by feasibility within the CIRCLE registry rather than by a prior power calculation. Because the reference distribution was derived from this sample, it is not a population-representative norm, and the threshold should be regarded as provisional until it is re-derived in an independent, population-representative sample. Future studies with larger, multicenter samples and formal sample size calculation are warranted. Second, the classification of MCI and NCI in this study relied on the NINDS-CSN battery rather than formal clinical diagnosis. Because the DCG and the NINDS-CSN battery assess overlapping cognitive domains, using the same battery both to define MCI/NCI status and to evaluate the DCG may introduce circularity. The ROC findings therefore support discrimination of battery-defined groups rather than clinical diagnostic accuracy, which may limit the interpretation of criterion validity. Third, the DCG was validated in a high vascular risk cohort but was not designed as a VCI-specific tool and did not undergo differential diagnosis against Alzheimer’s disease or mixed dementia. Validation in broader cognitive impairment populations is therefore needed. Fourth, only 43 participants returned for the second assessment. Although selection bias cannot be fully excluded, the retest subsample was randomly selected and did not differ significantly from non-retested participants on relevant baseline characteristics. Fifth, although cross-validation and bootstrap-based optimism correction suggested stable discriminative performance, the 12-item battery was derived and evaluated in the same sample, and independent external validation is required before it can be recommended for clinical or community use. Finally, we acknowledge that several factors that may influence test performance were not adequately measured, including prior tablet experience and motor limitations (hand dexterity, tremor, hemiparesis, and grip strength), which may affect performance on speed-based tasks.

## 5. Conclusions

The DCG battery may serve as a scalable, user-friendly tool for research-oriented cognitive screening and stratification, particularly in resource-limited settings where access to trained neuropsychological assessors is restricted. Because MCI/NCI status was defined by a neuropsychological battery rather than a formal clinical diagnosis, future studies should validate the DCG against clinical diagnostic standards before it is used for clinical screening or diagnosis.

## Figures and Tables

**Table 1:** DCG scores at baseline and 2-month follow-up, with practice effects and test–retest reliability (*n* = 43).

Digital Cognitive Games	M (SD) T1	M (SD) T2	t (T2 − T1) ^a^	*p* (T2 − T1)	BH-Adjusted *p* (T2 − T1)	Cohen’s d (T2 − T1) (95% CI)	r T1, T2 (95% CI)	ICC T1, T2 (95% CI)
Rapid Flash Memory	30.19 (34.79)	41 (35.58)	2.386	0.022 *	0.389	0.364 (0.053, 0.671)	0.644 (0.425, 0.791)	0.619 (0.339, 0.851)
Digit Span Memory	72.91 (35.11)	68.26 (30.04)	−1.524	0.135	1.000	−0.232 (−0.534, 0.072)	0.831 (0.708, 0.906)	0.808 (0.643, 0.908)
Spatial Card Matching	72.95 (30.24)	69.12 (26.67)	−1.389	0.172	1.000	−0.212 (−0.513, 0.092)	0.804 (0.665, 0.89)	0.795 (0.618, 0.897)
Visual Pattern Memory	55.79 (31.6)	62.07 (29.73)	2.459	0.018 *	0.326	0.375 (0.064, 0.682)	0.853 (0.742, 0.918)	0.836 (0.714, 0.917)
Speed Command Processing	63.4 (28.57)	62.05 (25.23)	−0.561	0.577	1.000	−0.086 (−0.385, 0.214)	0.836 (0.715, 0.908)	0.831 (0.644, 0.953)
Target Sum Challenge	83.7 (21.01)	86.65 (17.47)	1.305	0.199	1.000	0.199 (−0.104, 0.5)	0.717 (0.531, 0.837)	0.702 (0.392, 0.944)
Mental Arithmetic Speed	68.72 (36.29)	71.65 (31.99)	1.012	0.317	1.000	0.154 (−0.147, 0.454)	0.853 (0.743, 0.918)	0.846 (0.685, 0.952)
Parity Discrimination	90.35 (15.58)	92.09 (14.13)	0.982	0.332	1.000	0.15 (−0.152, 0.449)	0.696 (0.501, 0.824)	0.693 (0.35, 0.939)
Semantic Categorization	75.58 (24.88)	78.49 (24.66)	1.617	0.113	1.000	0.247 (−0.058, 0.549)	0.887 (0.799, 0.937)	0.883 (0.662, 1.000)
Lexical Classification	78.14 (21.98)	77.37 (22.44)	−0.376	0.709	1.000	−0.057 (−0.356, 0.242)	0.819 (0.687, 0.898)	0.822 (0.586, 0.951)
Spatial Memory Recall	63.58 (33.28)	60.49 (32.02)	−0.775	0.443	1.000	−0.118 (−0.417, 0.182)	0.679 (0.476, 0.814)	0.681 (0.367, 0.881)
Ripple Diffusion Tracking	64.37 (31.29)	66.67 (30.96)	0.893	0.377	1.000	0.136 (−0.165, 0.436)	0.852 (0.742, 0.918)	0.853 (0.568, 1.000)
Rapid Comparison	74.86 (26.78)	77.72 (25.28)	1.391	0.171	1.000	0.212 (−0.091, 0.513)	0.867 (0.767, 0.926)	0.863 (0.645, 0.988)
Fine Detail Detection	64.72 (32.16)	59.74 (30.59)	−1.446	0.156	1.000	−0.221 (−0.522, 0.083)	0.743 (0.569, 0.853)	0.737 (0.526, 0.871)
Angles	74.49 (25.09)	72.88 (29.42)	−0.436	0.665	1.000	−0.067 (−0.365, 0.233)	0.618 (0.391, 0.775)	0.615 (0.29, 0.883)
Mystery Garden	38.91 (33.55)	38.37 (31.03)	−0.178	0.859	1.000	−0.027 (−0.326, 0.272)	0.817 (0.685, 0.897)	0.818 (0.696, 0.898)
Avian Visual Discrimination	42.63 (32.18)	45.02 (30.73)	0.826	0.414	1.000	0.126 (−0.175, 0.425)	0.818 (0.686, 0.898)	0.818 (0.634, 0.922)
Face-Name Association task	60.47 (32.84)	62.84 (30.28)	0.845	0.403	1.000	0.129 (−0.172, 0.428)	0.833 (0.71, 0.906)	0.831 (0.566, 0.986)

^a^ Paired-samples *t*-test were used to compare T1 and T2 scores (df = 42). *, *p* < 0.05. M, mean; SD, standard deviation; d, Cohen’s d.

**Table 2:** Pearson correlation of cognitive scores between digital games and paper-and-pencil NINDS-CSN tests (*n* = 86).

	Complete Version of DCG		Reduced Version of DCG	
Cognitive Domains	Correlation	*p* Value (B-H)	Correlation	*p* Value (B-H)
Attention	0.460	<0.001	0.460	<0.001
Executive function	0.559	<0.001	0.572	<0.001
Language	0.373	0.007	0.389	0.004
Visuomotor speed	0.582	<0.001	0.534	<0.001
Visuospatial function	0.260	0.063	0.320	0.023
Memory	0.490	<0.001	0.490	<0.001
Global cognition	0.734	<0.001	0.751	<0.001

DCG, digital cognitive assessment game.

**Table 3:** Test battery canonical structure correlations (*n* = 86).

Digital Cognitive Games	Correlation	Paper-and-Pencil Test Battery	Correlation
Rapid Flash Memory (Visuomotor speed)	0.6769	Color Trail Test B (Executive function)	0.7793
Parity Discrimination (Executive function)	0.6297	Symbol Digit Modalities Test (Visuomotor speed)	0.7563
Rapid Comparison (Visuomotor speed)	0.6005	Digit Span Backward (Attention)	0.6898
Lexical Classification (Language)	0.5962	Hopkins Verbal Learning Test Immediate Recall (Memory)	0.6761
Face-Name Association Task (Memory)	0.5824	Verbal Fluency (Executive function)	0.6457
Fine Detail Detection (Visuomotor speed)	0.5488	Rey Complex Figure Test Copy (Visuospatial function)	0.6260
Target Sum Challenge (Attention)	0.5427	Color Trail Test A (Executive function)	0.5860
Digit Span Memory (Attention)	0.5133	Rey Complex Figure Test Delayed Recall (Memory)	0.5322
Semantic Categorization (Language)	0.5045	Rey Complex Figure Test Immediate Recall (Memory)	0.5308
Visual Pattern Memory (Executive function)	0.4867	Hopkins Verbal Learning Test Recognition (Memory)	0.5278
Speed Command Processing (Executive function)	0.4772	Modified Boston Naming Test (Language)	0.4700
Avian Visual Discrimination (Memory)	0.4710	Hopkins Verbal Learning Test Delayed Recall (Memory)	0.4390
Spatial Card Matching (Executive function)	0.4663	Rey Complex Figure Test Recognition (Memory)	0.4263
Mystery Garden (Visuospatial function)	0.4460	Digit Span Forward (Attention)	0.4183
Ripple Diffusion Tracking (Visuomotor speed)	0.3636		
Angles (Visuospatial function)	0.2927		
Spatial Memory Recall (Visuomotor speed)	0.2529		
Mental Arithmetic Speed (Executive function)	0.1981		

## Data Availability

The data presented in this study are available on request from the corresponding author (the data are not publicly available due to privacy and ethical restrictions).

## Supplementary Materials

The following supporting information can be downloaded at: https://www.mdpi.com/article/10.3390/brainsci16101125/s1, Table S1. Tasks of the Digital Cognitive Games and conventional paper-based assessments and their corresponding cognitive domains; Table S2. Baseline Characters of Participants; Table S3. ICCs for domain and global cognition score of DCG; Table S4. Pearson correlation of cognitive scores between DCG items and paper-and-pencil NINDS-CSN test (*n* = 86); Table S5. The confusion matrix for the 18-item DCG and 12 item DCG; Table S6. Results of diagnostic performance of the DCG on MCI; Table S7. Pearson correlation of cognitive scores between DCG items or paper-and-pencil NINDS-CSN test and education years (*n* = 86); Table S8. Exclusion category and rationale for tasks removed from the 12-item battery; Table S9. Spearman correlation of user-experience rating and DCG task performance (*n* = 86); Figure S1. Participant flowchart, and supplemental methods.

## Abbreviations

The following abbreviations are used in this manuscript:

AUC	Area under the curve
CCA	Canonical correlation analysis
DCG	Digital Cognitive Assessment Game
MCI	Mild cognitive impairment
MMSE	Mini-Mental State Examination
MoCA	Montreal Cognitive Assessment
NINDS-CSN	National Institute of Neurological Disorders and Stroke and Canadian Stroke Network
ICC	Intraclass correlation coefficient
