# Supplementary material for: Reliability and Validity of a Gamified Cognitive Assessment for Adults at High Risk of Stroke
Source: Brain Sci. 2026 Oct 18;16(10):1125. doi: 10.3390/brainsci16101125 (PMC13702261; doi:10.3390/brainsci16101125)

Supplementary Material

Materials and Methods

The DCG was developed in four stages:

(1) **Conceptualization and needs assessment.** We reviewed published literature on digital cognitive assessment and vascular cognitive impairment, and consulted with clinicians and researchers experienced in cognitive assessment of older adults. This stage identified the need for a self-administered, domain-specific, gamified tool suitable for older Chinese adults at high vascular risk.

(2) **Task design and domain mapping.** The battery covers six cognitive domains: attention, executive function, language, visuomotor speed, visuospatial function, and memory. Among these, executive function and visuomotor speed were prioritized, as they are the domains most commonly affected in vascular cognitive impairment, and they contain the largest number of tasks in the battery. Domain selection was guided by the NINDS-CSN vascular cognitive impairment harmonization standards and a narrative review of digital cognitive assessment and gamified cognitive training. Eighteen gamified tasks were designed or adapted; task paradigms were adapted from previously developed cognitive training games from our group and from published digital assessment studies.

(3) **Prototype development and iterative refinement.** Initial prototypes were developed for tablet use, with simplified interfaces, large touch targets, and audio-visual instructions tailored to older adults. The prototypes underwent iterative refinement based on feedback from around 20 pilot users and research staff, including adjustments to instruction clarity, task difficulty, and interface layout.

(4) **Pilot usability testing and finalization.** A pilot sample of older adults completed early versions of the battery. Their feedback informed further modifications, including cultural adaptation of stimuli (e.g., Chinese characters and faces in the Face – Name Association Task) and simplification of task instructions. The final 18-task version was then used in the present validation study.

Table S1 Tasks of the Digital Cognitive Games and conventional paper-based assessments and their corresponding cognitive domains

Cognitive Domain	Neuropsychological tests	Digital Cognitive games	Score for DCG
Attention	Digit Span Forward	Digit Span Memory	Accuracy rate
	Digit Span Backward	Target Sum Challenge	Accuracy rate
Executive function	Verbal Fluency	Speed Command Processing	Reaction time
	Color Trail Test A	Visual Pattern Memory	Reaction time
	Color Trail Test B	Mental Arithmetic Speed	Reaction time
		Parity Discrimination	Reaction time
		Spatial Card Matching	Reaction time
Language	15-item Modified Boston Naming Test	Semantic Categorization	Accuracy rate
		Lexical Classification	Accuracy rate
Visuomotor speed	Symbol Digit Modalities Test	Ripple Diffusion Tracking	Accuracy rate
		Rapid Flash Memory	Accuracy rate
		Rapid Comparison	Accuracy rate
		Spatial Memory Recall	Accuracy rate
		Fine Detail Detection	Accuracy rate
Visuospatial function	Rey Complex Figure Test Copy	Angles	Accuracy rate
		Mystery Garden	Accuracy rate
Memory	Rey Complex Figure Test Immediate Recall	Avian Visual Discrimination	Accuracy rate
	Rey Complex Figure Test Delayed Recall	Face-Name Association Task	Accuracy rate
	Rey Complex Figure Test Recognition		
	Hopkins Verbal Learning Test Delayed Recall		
	Hopkins Verbal Learning Test Immediate Recall		
	Hopkins Verbal Learning Test Recognition		

Table S2 Baseline Characters of Participants

Characters	Total（*n*=86）	Non-retest（*n*=43）	retest（*n*=43）	P
Age,Mean ± SD	67.38 ± 7.06	67.12 ± 7.87	67.65 ± 6.23	0.728
Female,N(%)	56 (65.1%)	28 (65.1%)	28 (65.1%)	1.000
Education Grade,N(%)				0.580
≤6th grade	13 (15.1%)	6 (14.0%)	7 (16.3%)	
6-9th grade	25 (29.1%)	12 (27.9%)	13 (30.2%)	
High school graduate	24 (27.9%)	10 (23.3%)	14 (32.6%)	
≥ College graduate	24 (27.9%)	15 (34.9%)	9 (20.9%)	
Education Year,Mean ± SD	11.37 ± 3.56	11.79 ± 3.76	10.95 ± 3.33	0.278
BMI,Mean ± SD	24.33 ± 3.03	23.87 ± 2.68	24.80 ± 3.32	0.158
Smoking,N(%)	14 (16.3%)	9 (20.9%)	5 (11.6%)	0.381
Drinking,N(%)	16 (18.6%)	9 (20.9%)	7 (16.3%)	0.782
Lack of exercise,N(%)	53 (61.6%)	27 (62.8%)	26 (60.5%)	1.000
Cerebro vascular Disease,N(%)	8 (9.3%)	5 (11.6%)	3 (7.0%)	0.710
Prior Stroke	5 (5.8%)	4 (9.3%)	1 (2.3%)	0.357
Prior TIA	3 (3.5%)	1 (2.3%)	2 (4.7%)	1.000
Heart Disease,N(%)	10 (11.6%)	3 (7%)	7 (16.3%)	0.313
Hypertention,N(%)	66 (76.7%)	30 (69.8%)	36 (83.7%)	0.202
Hyperlipidemia,N(%)	40 (46.5%)	19 (44.2%)	21 (48.8%)	0.829
Diabetes,N(%)	20 (23.3%)	12 (27.9%)	8 (18.6%)	0.444
Depression score[a],Mean ± SD	1.51 ± 3.29	1.09 ± 2.59	1.93 ± 3.85	0.241
Anxiety score[b],Mean ± SD	0.99 ± 2.35	0.74 ± 2.17	1.23 ± 2.52	0.339
CMMS[c],Mean ± SD	27.43 ± 2.45	27.56 ± 2.49	27.30 ± 2.43	0.631
MoCA[d],Mean ± SD	23.86 ± 4.52	23.88 ± 4.70	23.84 ± 4.40	0.962

a, depression score was assessed with Hamilton depressive scale,

b, anxiety score was assessed with Hamilton anxiety scale

c, Chinese Mini-Mental Status

d, Montreal Cognitive Assessment

Table S3 ICCs for domain and global cognition score of DCG

Cognitive domains	ICC	Lower	Upper	P
Attention	0.805	0.666	0.889	<0.001
Executive function	0.893	0.812	0.941	<0.001
Language	0.894	0.814	0.941	<0.001
Visuomotor speed	0.85	0.74	0.915	<0.001
Visuospatial function	0.793	0.649	0.883	<0.001
Memory	0.904	0.83	0.947	<0.001
Global cognition	0.901	0.826	0.945	<0.001

Table S4 Pearson correlation of cognitive scores between DCG items and paper-and-pencil NINDS-CSN test（*n*=86）

DCG/Paper-and-pencil	**Attention**	**Executive function**	**Language**	**Visuomotor speed**	**Visuospatial function**	**Memory**	**Global cognition**
Digit Span Memory	0.336(**)	0.317(**)	0.319(**)	0.400(***)	0.377(***)	0.392(***)	0.471(***)
Target Sum Challenge	0.398(***)	0.379(***)	0.331(**)	0.306(**)	0.266(*)	0.320(**)	0.44(***)
Speed Command Processing	0.330(**)	0.387(***)	0.325(**)	0.390(***)	0.151	0.458(***)	0.449(***)
Visual Pattern Memory	0.231(*)	0.389(***)	0.167	0.334(**)	0.376(***)	0.387(***)	0.414(***)
Mental Arithmetic Speed	0.083	0.194	0.341(**)	0.164	0.177	0.312(**)	0.279(**)
Parity Discrimination	0.309(**)	0.528(***)	0.394(***)	0.390(***)	0.445(***)	0.468(***)	0.557(***)
Spatial Card Matching	0.291(**)	0.426(***)	0.418(***)	0.378(***)	0.229(*)	0.421(***)	0.476(***)
Lexical Classification	0.427(***)	0.446(***)	0.389(***)	0.312(**)	0.372(***)	0.328(**)	0.500(***)
Semantic Categorization	0.251(*)	0.298(**)	0.288(**)	0.310(**)	0.387(***)	0.467(***)	0.440(***)
Ripple Diffusion Tracking	0.188	0.273(*)	0.237(*)	0.460(***)	0.072	0.153	0.304(**)
Rapid Flash Memory	0.388(***)	0.44(***)	0.220(*)	0.480(***)	0.313(**)	0.377(***)	0.488(***)
Rapid Comparison	0.409(***)	0.433(***)	0.322(**)	0.362(***)	0.414(***)	0.420(***)	0.519(***)
Spatial Memory Recall	0.168	0.192	0.171	0.226(*)	0.037	0.261(*)	0.232(*)
Fine Detail Detection	0.210	0.416(***)	0.271(*)	0.405(***)	0.324(**)	0.374(***)	0.440(***)
Angles	0.111	0.231(*)	0.215(*)	0.239(*)	0.125	0.254(*)	0.258(*)
Mysterious Garden	0.244(*)	0.397(***)	0.149	0.298(**)	0.320(**)	0.262(*)	0.368(***)
Face-Name Association Task	0.370(***)	0.294(**)	0.301(**)	0.250(*)	0.287(**)	0.398(***)	0.417(***)
Avian Visual Discrimination	0.205	0.400(***)	0.109	0.291(**)	0.182	0.376(***)	0.344(**)

(*): P <0.05, (**): P <0.01, (***): P <0.001

Table S5 The confusion matrix for the 18-item DCG and 12 item DCG

	MCI	NCI
18-item DCG (cut-off: global z < 0.22)		
DCG positive	26	14
DCG negative	1	45
12 item DCG (cut-off: global z < 0.014)		
DCG positive	25	10
DCG negative	2	49

Table S6 Results of diagnostic performance of the DCG on MCI[a]

DCG version	Cut-off	AUC(95%CI)	Sensitivity(95%CI)	Specificity(95%CI)	PPV[b](95%CI)	NPV[c](95%CI)
18-item version	0.22	0.91(0.85-0.97)	0.96(0.82–0.99)	0.76(0.64–0.85)	0.65(0.50–0.78)	0.98(0.89–1.00)
12-item version	0.01	0.91(0.85-0.97)	0.93(0.77-0.98)	0.83(0.72-0.91)	0.71(0.55-0.84)	0.96(0.87-0.99)

a,MCI, mild cognitive impairment; b,PPV, positive predictive value; c,NPV, negative predictive value.

Table S7 Pearson correlation of cognitive scores between DCG items or paper-and-pencil NINDS-CSN test and education years（*n*=86）

DCG	r	BH-adjusted P	paper-and-pencil NINDS-CSN test	r	BH-adjusted P
Spatial Memory Recall	0.255	0.322	Digit Span Forward	0.301	0.068
Ripple Diffusion Tracking	0.148	1.000	Digit Span Backward	0.303	0.064
Avian Visual Discrimination	0.247	0.392	Verbal Fluency	0.453	<0.001
Speed Command Processing	0.303	0.083	Color Trail Test A	0.143	1.000
Target Sum Challenge	0.155	1.000	Color Trail Test B	0.308	0.055
Rapid Comparison	0.220	0.760	Modified Boston Naming Test	0.509	<0.001
Fine Detail Detection	0.137	1.000	Symbol Digit Modalities Test	0.368	0.007
Angles	0.142	1.000	Rey Complex Figure Test Copy	0.185	1.000
Mystery Garden	0.122	1.000	Hopkins Verbal Learning Test Delayed Recall	0.238	0.379
Semantic Categorization	0.375	0.007	Rey Complex Figure Test Immediate Recall	0.303	0.063
Mental Arithmetic Speed	0.075	1.000	Rey Complex Figure Test Delayed Recall	0.376	0.005
Rapid Flash Memory	0.270	0.212	Rey Complex Figure Test Recognition	-0.017	1.000
Face-Name Association Task	0.364	0.010	Hopkins Verbal Learning Test Immediate Recall	0.323	0.034
Lexical Classification	0.402	0.002	Hopkins Verbal Learning Test Recognition	0.297	0.076
Digit Span Memory	0.279	0.165			
Spatial Card Matching	0.247	0.396			
Parity Discrimination	0.364	0.010			
Visual Pattern Memory	0.249	0.376			

Table S8 Exclusion category and rationale for tasks removed from the 12-item battery

Task	Exclusion category	Rationale
Rapid Flash Memory	Reliability + practice effect	ICC = 0.619 (95% CI: 0.339–0.851); moderate raw practice effect (Cohen's d = 0.364)
Visual Pattern Memory	Practice effect	Moderate raw practice effect (Cohen's d = 0.375)
Mental Arithmetic Speed	Validity	Weak convergent validity with paper-and-pencil measures in the corresponding cognitive domain (r = 0.194)
Semantic Categorization	Validity	Weak convergent validity with paper-and-pencil measures in the corresponding cognitive domain (r = 0.288)
Angles	Validity	Weak convergent validity (r = 0.125)
Spatial Memory Recall	Usability + validity	Low understandability of instructions (13% disagree that instructions were easy to understand); weak convergent validity (r = 0.226)

Table S9 Spearman correlation of user-experience rating and DCG task performance (n=86)

Game	Instruction Understandability[a]		Difficulty[b]		Enjoyment[c]		Anxiety[d]		Concentration[e]	
	rho	P (BH)	rho	P (BH)	rho	P (BH)	rho	P (BH)	rho	P (BH)
Digit Span Memory	-0.018	0.866	-0.032	0.916	-0.056	0.758	-	-	-	-
Target Sum Challenge	0.069	0.658	-0.060	0.916	0.178	0.358	-	-	-	-
Speed Command Processing	0.051	0.670	-0.006	0.960	0.163	0.417	-	-	-	-
Visual Pattern Memory	0.100	0.644	-0.186	0.399	-0.236	0.143	-	-	-	-
Mental Arithmetic Speed	-0.087	0.658	-0.059	0.916	-0.079	0.715	-	-	-	-
Parity Discrimination	0.188	0.310	0.014	0.938	0.049	0.779	-	-	-	-
Spatial Card Matching	0.061	0.658	-0.325	0.056	-0.031	0.839	-	-	-	-
Semantic Categorization	0.102	0.644	-0.112	0.637	0.075	0.715	-	-	-	-
Lexical Classification	0.216	0.310	-0.163	0.416	0.239	0.143	-	-	-	-
Ripple Diffusion Tracking	0.116	0.601	-0.065	0.916	0.073	0.715	-	-	-	-
Rapid Flash Memory	0.184	0.310	-0.141	0.468	0.078	0.715	-	-	-	-
Rapid Comparison	0.195	0.310	-0.034	0.916	0.126	0.587	-	-	-	-
Spatial Memory Recall	-0.056	0.658	0.044	0.916	0.085	0.715	-	-	-	-
Fine Detail Detection	0.072	0.658	-0.054	0.916	-0.027	0.839	-	-	-	-
Angles	0.179	0.310	-0.037	0.916	-0.071	0.715	-	-	-	-
Mystery Garden	0.064	0.658	0.035	0.916	0.019	0.863	-	-	-	-
Avian Visual Discrimination	0.214	0.310	-0.026	0.927	0.145	0.506	-	-	-	-
Face-Name Association Task	0.083	0.658	-0.176	0.399	0.320	0.053	-	-	-	-
Attention	0.064	0.658	-0.149	0.468	0.123	0.587	-0.061	0.577	0.039	0.839
Executive function	0.071	0.658	-0.243	0.199	-0.063	0.747	-0.147	0.336	0.022	0.839
Language	0.142	0.481	-0.194	0.399	0.269	0.103	-0.093	0.551	0.055	0.839
Visuomotor speed	0.143	0.481	-0.138	0.468	0.041	0.803	-0.183	0.321	-0.031	0.839

Visuospatial function	0.124	0.583	-0.018	0.938	-0.103	0.715	-0.064	0.577	-0.078	0.839
Memory	0.225	0.310	-0.173	0.399	0.306	0.053	-0.142	0.336	-0.055	0.839
global cognition	0.192	0.310	-0.248	0.199	0.195	0.299	-0.222	0.282	-0.041	0.839

Values are Spearman's rho with BH-adjusted P values. Task-level items (understandability, difficulty, enjoyment) were correlated with the corresponding game ,domain and global DCG performance; overall items (anxiety, concentration) were correlated with domain and global DCG performance. BH, Benjamini-Hochberg; DCG, Digital Cognitive Assessment Games.

a, Understandability, agreement score for "the instructions were easy to understand" for each game;

b, Difficulty, agreement score for "the game was difficult" for each game;

c, Enjoyment, agreement score for "I enjoyed the game" for each game;

d, Anxiety, agreement score for "I felt anxious about my performance" for the DCG overall.

e,Concentration, agreement score for "I found it difficult to stay focused" for the DCG overall.

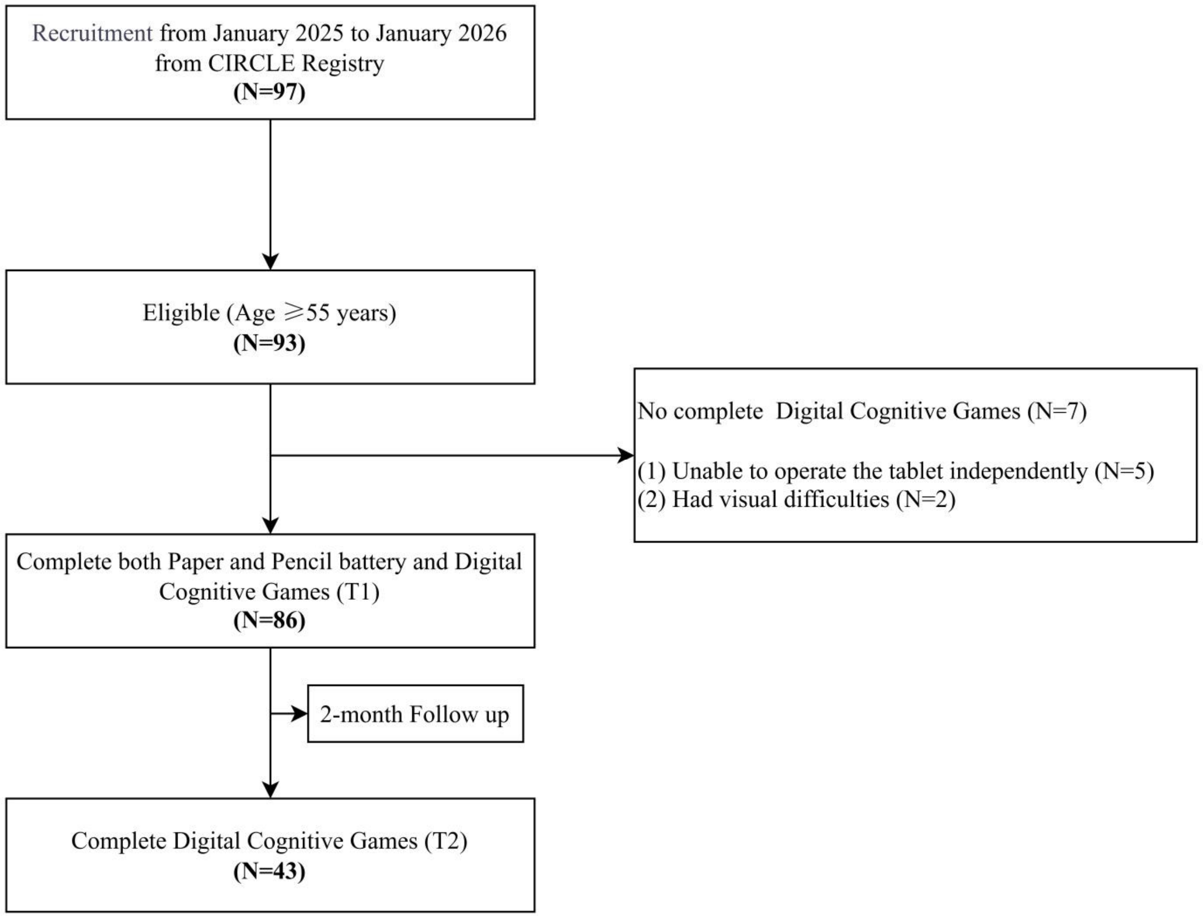

Figure S1. Participant flowchart. T1, baseline; T2, follow-up (2 months after complete baseline).

Rules of DCG

Rapid Flash Memory: A honeycomb pattern composed of 19 hexagonal cells will be presented on the screen. A subset of these cells will be highlighted in orange for a brief duration. Participants are required to encode and retain the spatial locations of the highlighted (orange) hexagons. Following the disappearance of the highlighted cues, participants must identify and select the previously highlighted hexagons by tapping on their corresponding positions within the grid.

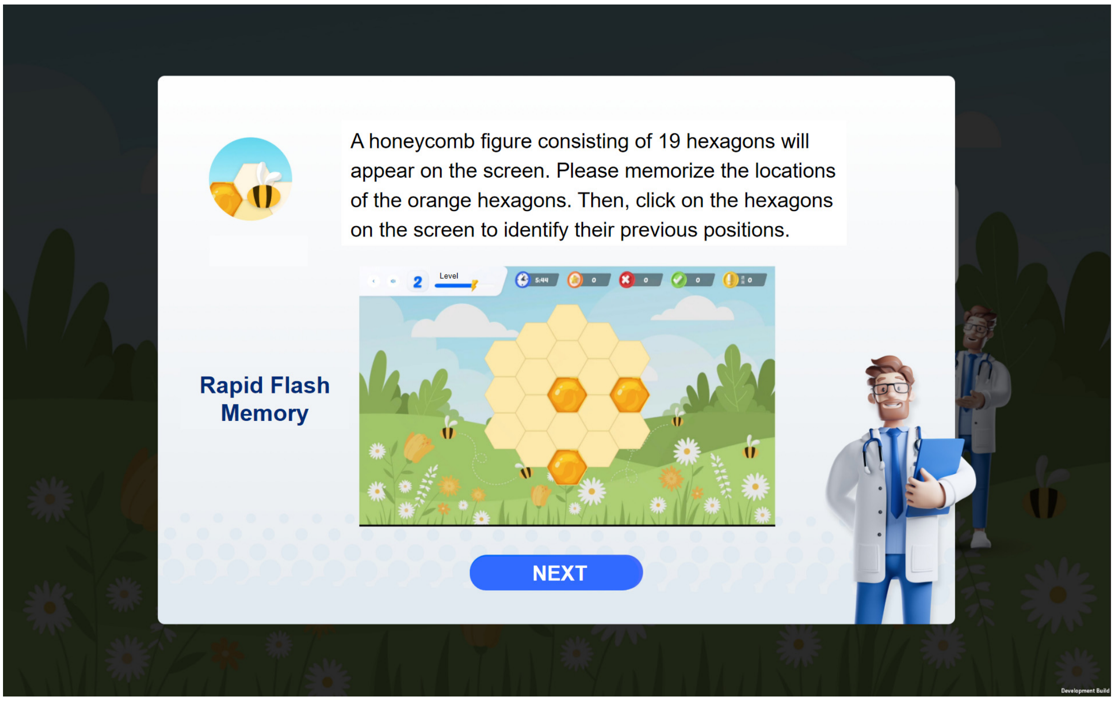

Digit Span Memory: A sequence of digits will be presented sequentially on the screen. Participants are required to encode and retain all presented digits in the order of appearance. Upon completion of the presentation phase, participants must identify the digits that were previously displayed and reconstruct the sequence by selecting them in the exact order in which they appeared.

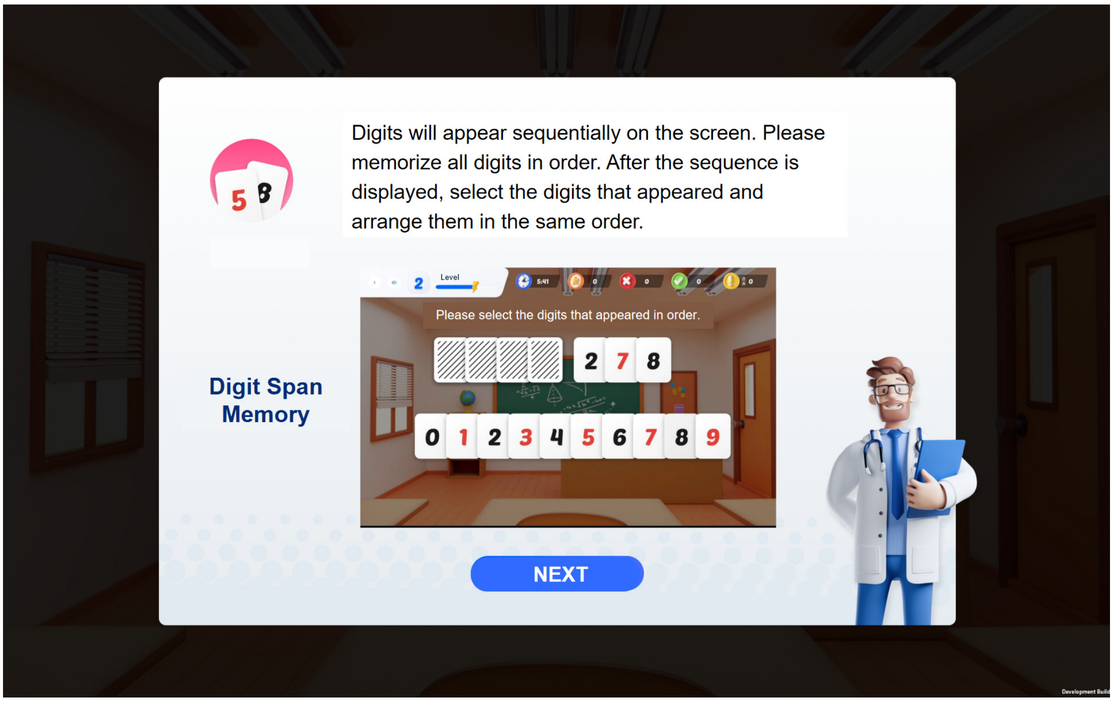

Spatial Card Matching: Colored blocks will be presented in the center of the screen, with a subset of blocks highlighted in orange. Participants are required to encode and retain the spatial positions of the orange-colored blocks during the presentation phase. Subsequently, all blocks will be flipped, removing the color cues. Participants must then identify and select the positions corresponding to the previously presented orange blocks.

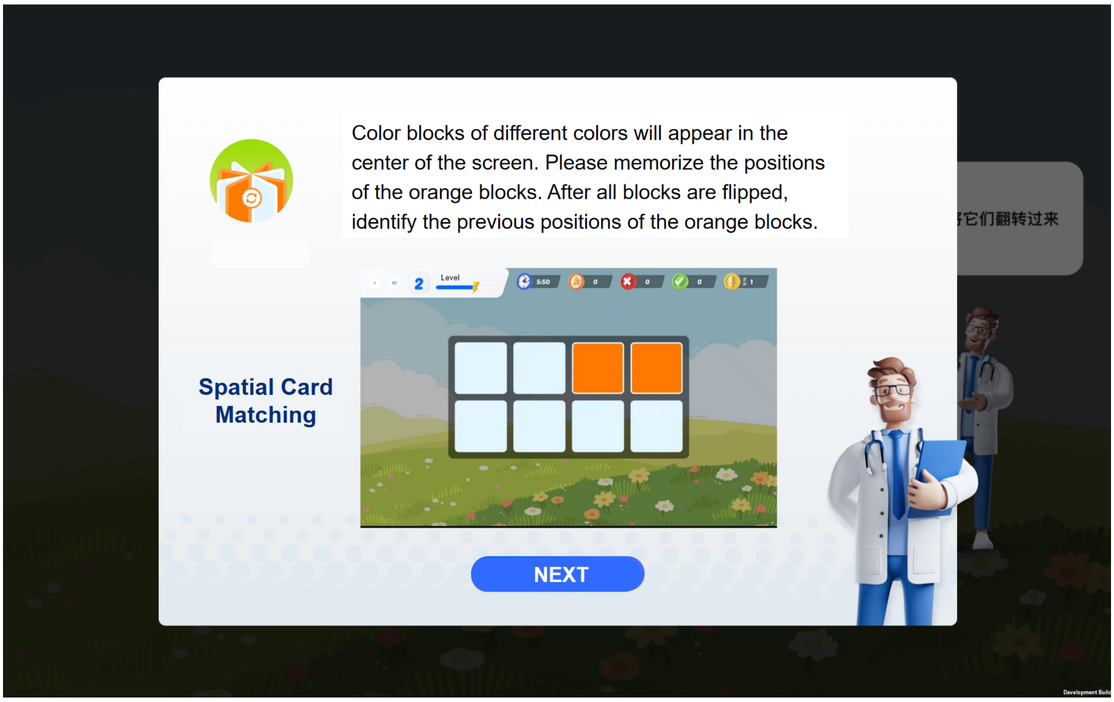

Visual Pattern Memory: Four groups of animal images will be presented at the center of the screen. Participants are required to encode and retain the spatial locations of these images during the initial presentation phase. Subsequently, all images will be flipped and concealed. Participants must then identify and match pairs of identical images by selecting two corresponding positions at a time.

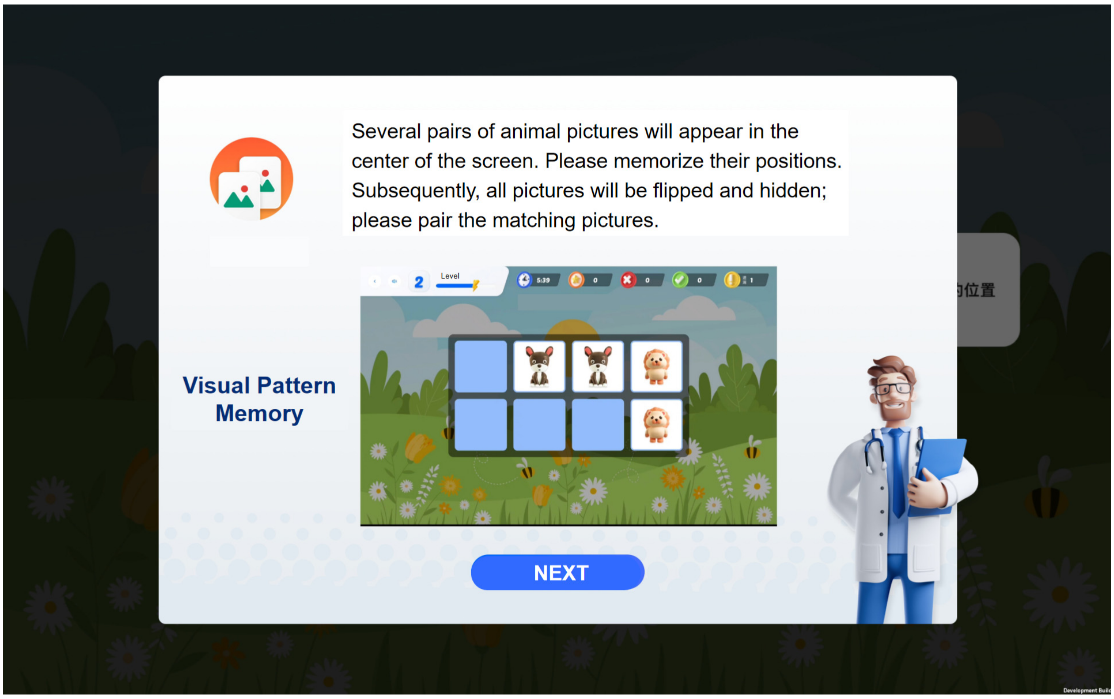

Speed Command Processing: Four hand icons indicating the four cardinal directions (up, down,

left, right) are displayed on the screen. A central textual cue specifies a direction, along with an instruction indicating whether the response should be congruent (same) or incongruent (opposite) to the cue. Participants must select the hand icon corresponding to the correct direction based on the given rule. Responses are scored as correct only if they satisfy the instructed relationship (congruent or incongruent) with the central cue.

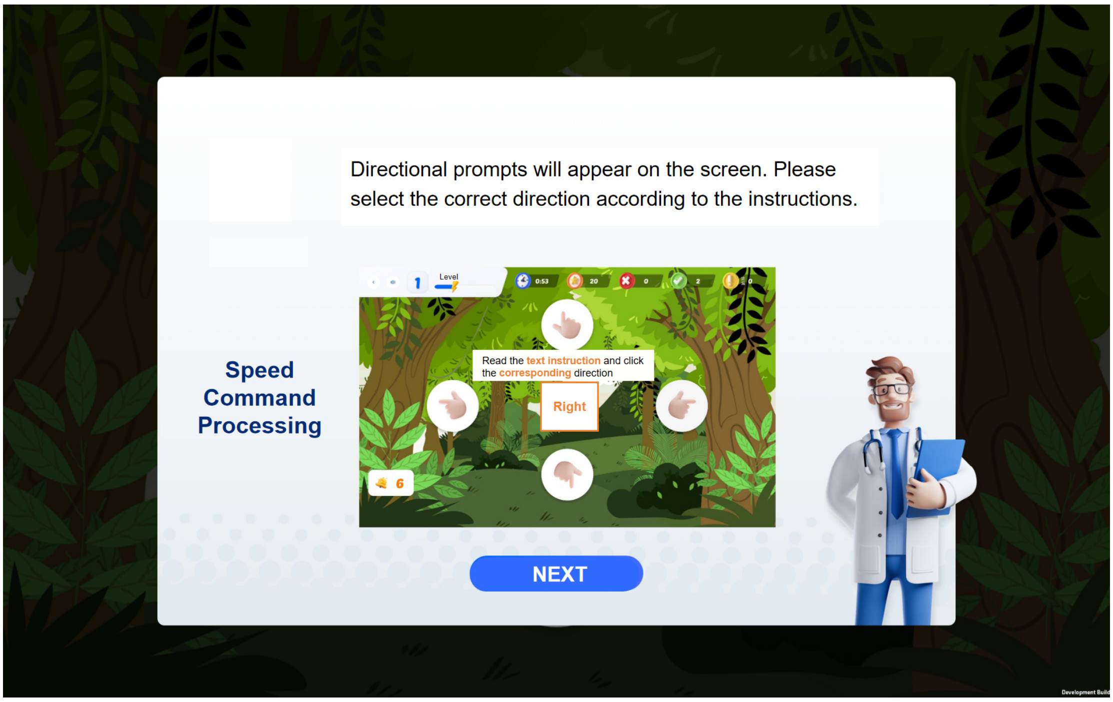

Target Sum Challenge: A target number is presented at the top of the screen. Participants are required to select one or more numerical cards from a set displayed below such that the sum of the selected numbers equals the target value. Responses are considered correct only when the selected combination yields the exact target number.

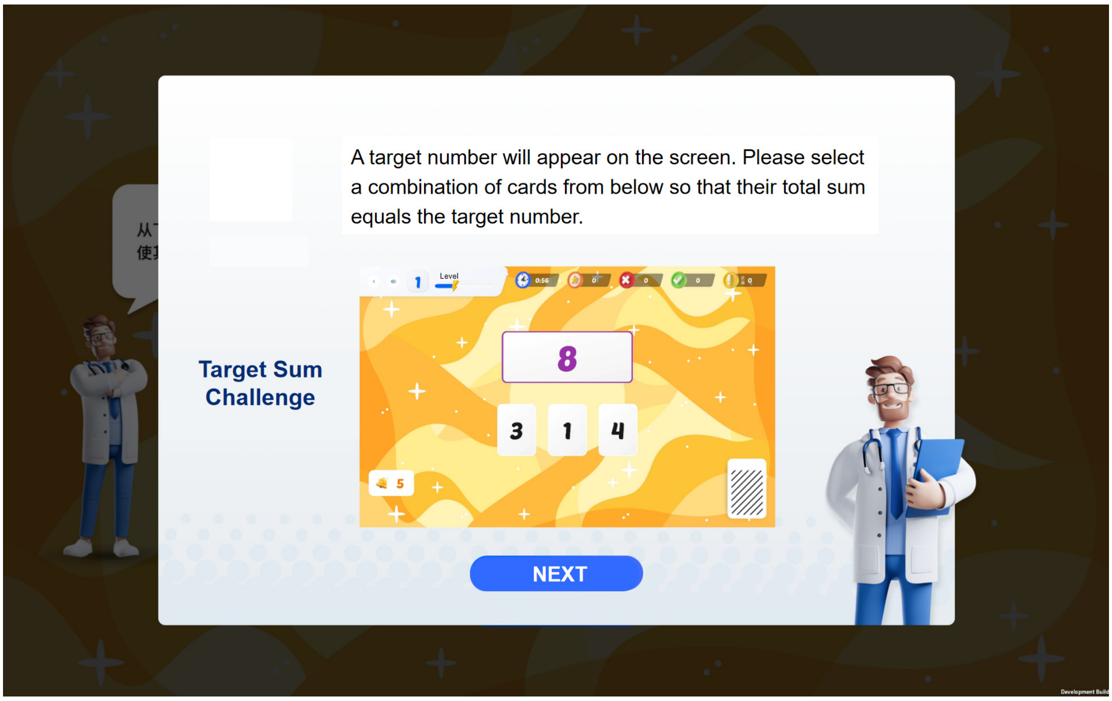

Mental Arithmetic Speed: A number of oranges will be displayed on the screen, each labeled with a randomly assigned digit (1-9). Participants are required to determine the total count of digits presented across all oranges and select the corresponding number from the set of options provided below.

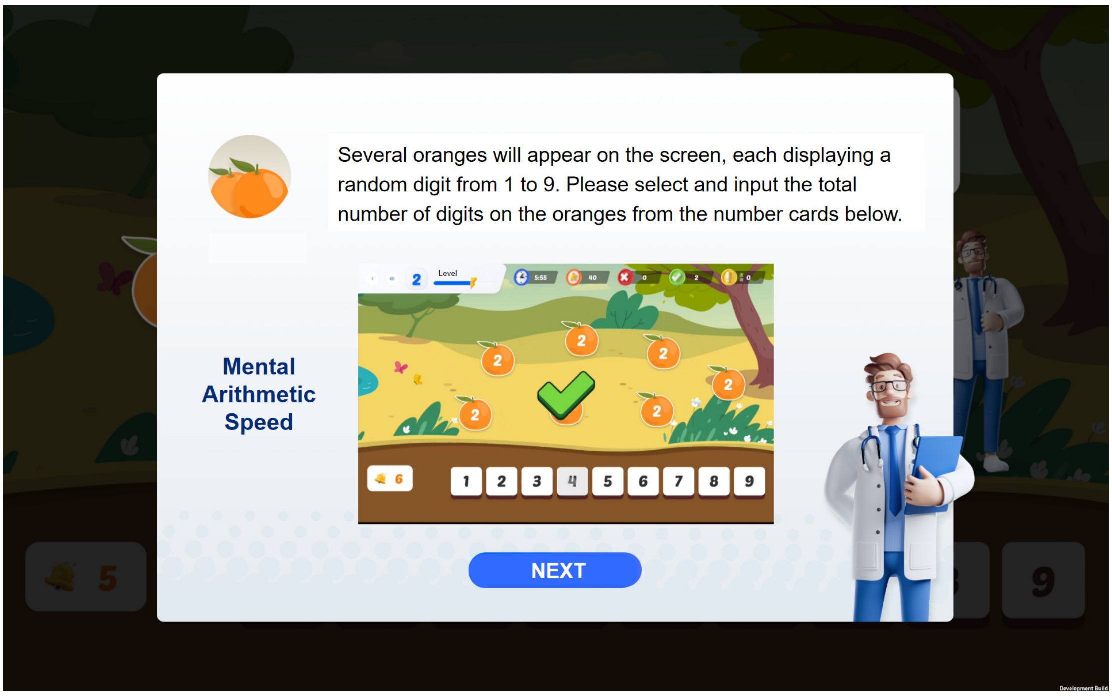

Parity Discrimination: Numbers ranging from 1 to 99 will be displayed on the screen. Participants are required to judge whether each number is odd or even. If the number is odd, they must press the "odd number" button; if the number is even, they must press the "even number" button.

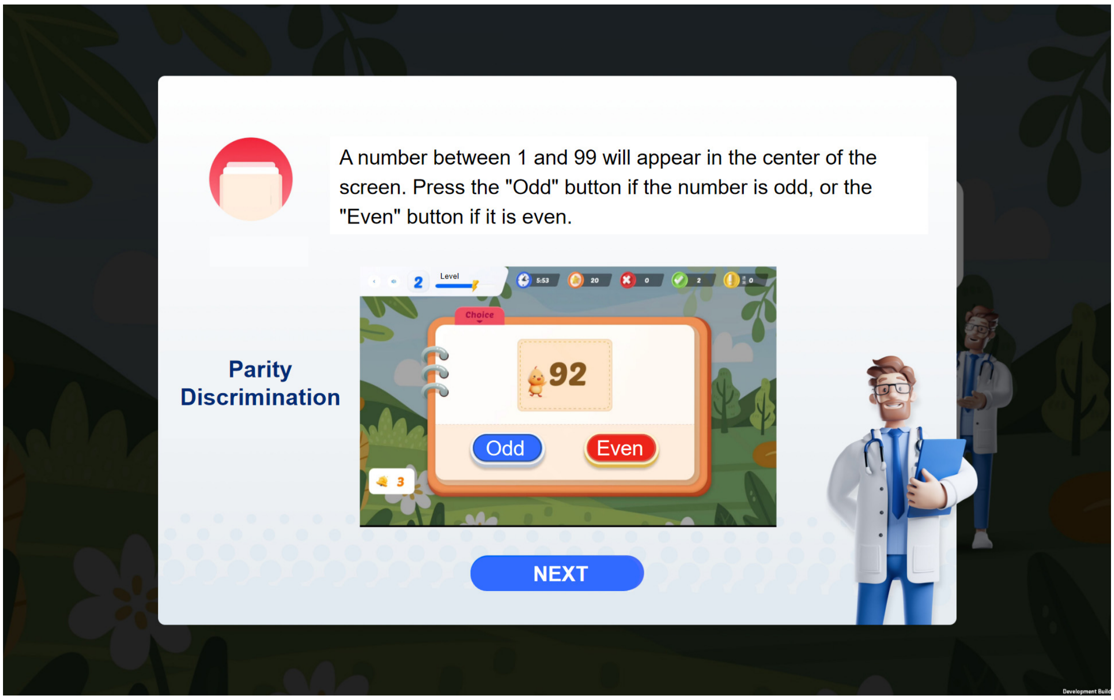

Semantic Categorization: Two words will be presented on the screen. Participants are required to determine whether the two words belong to the same semantic category.

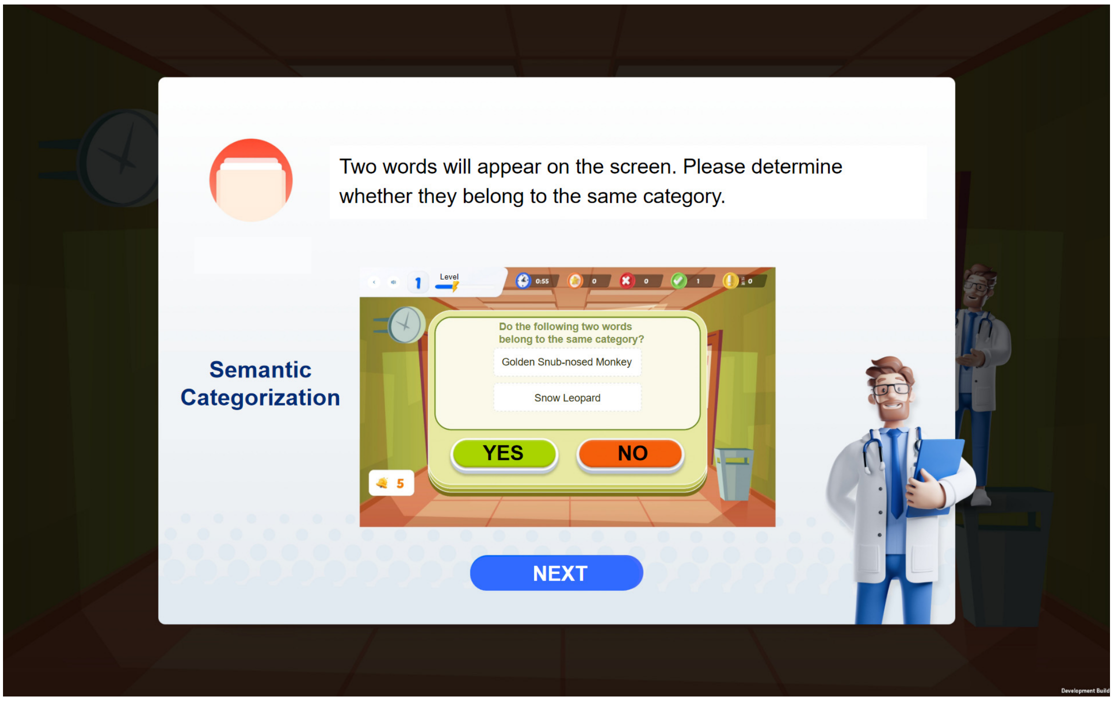

Lexical Classification: A single word will be presented on the left side of the screen. Participants are required to categorize the word into one of three semantic classes: Animal, Fruit, or Color, by selecting the corresponding category from the options displayed on the right.

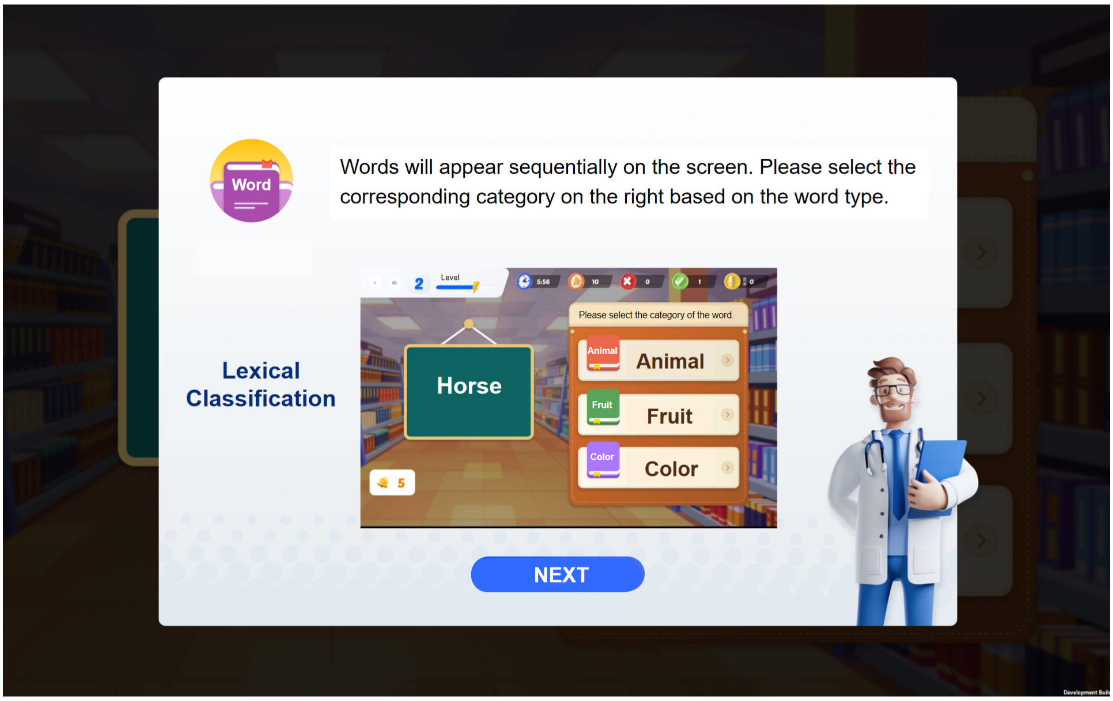

Spatial Memory Recall: Two target balls are initially presented on the screen. These balls move randomly, while additional identical balls gradually appear and move along similar trajectories.After all motion ceases, participants are required to identify the two original target balls.

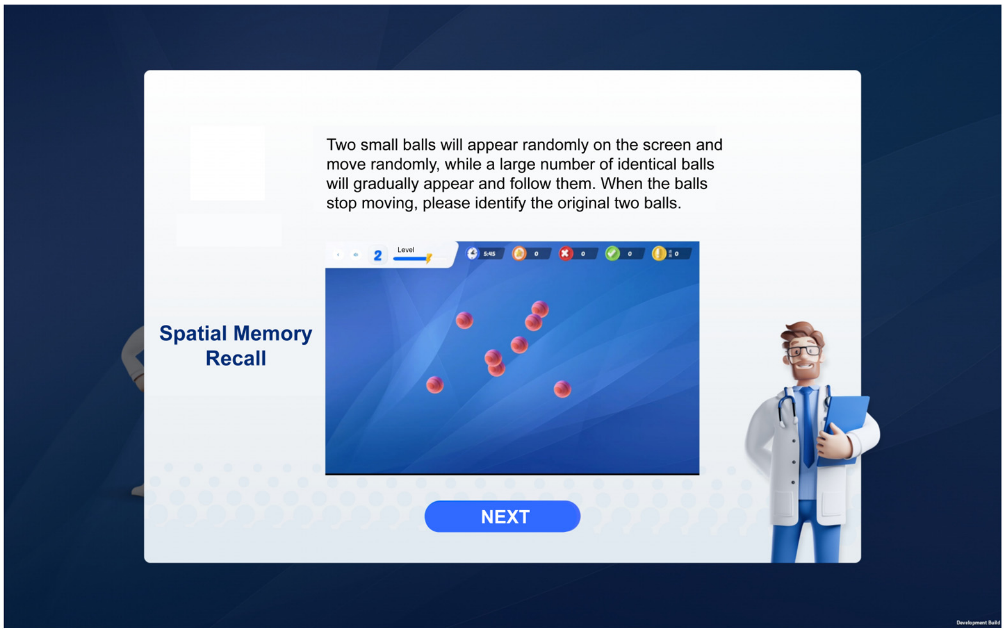

Ripple Diffusion Tracking: A circular wave pattern will be presented on the screen, exhibiting rapid dynamic fluctuations. Participants are required to determine the direction of the wave motion, specifically whether it propagates to the inward or the outward.

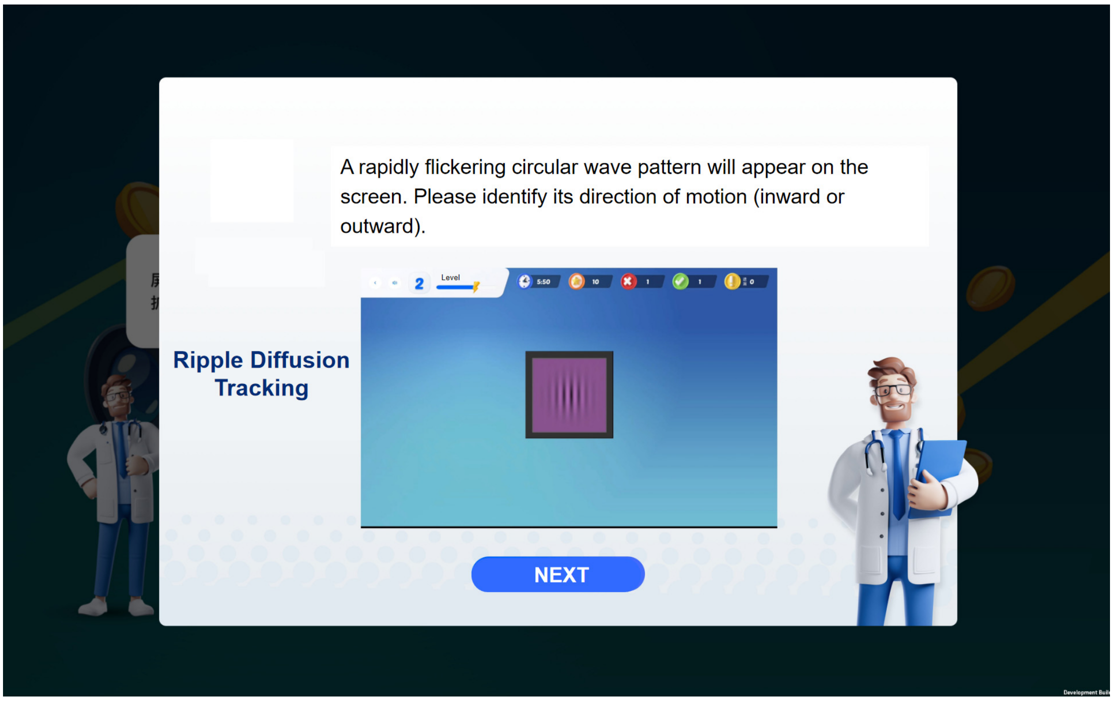

Rapid Comparison: Two dot-pattern stimuli, which are either highly similar or identical, are presented simultaneously on the screen. Participants are required to rapidly determine whether the left and right patterns are the same or different.

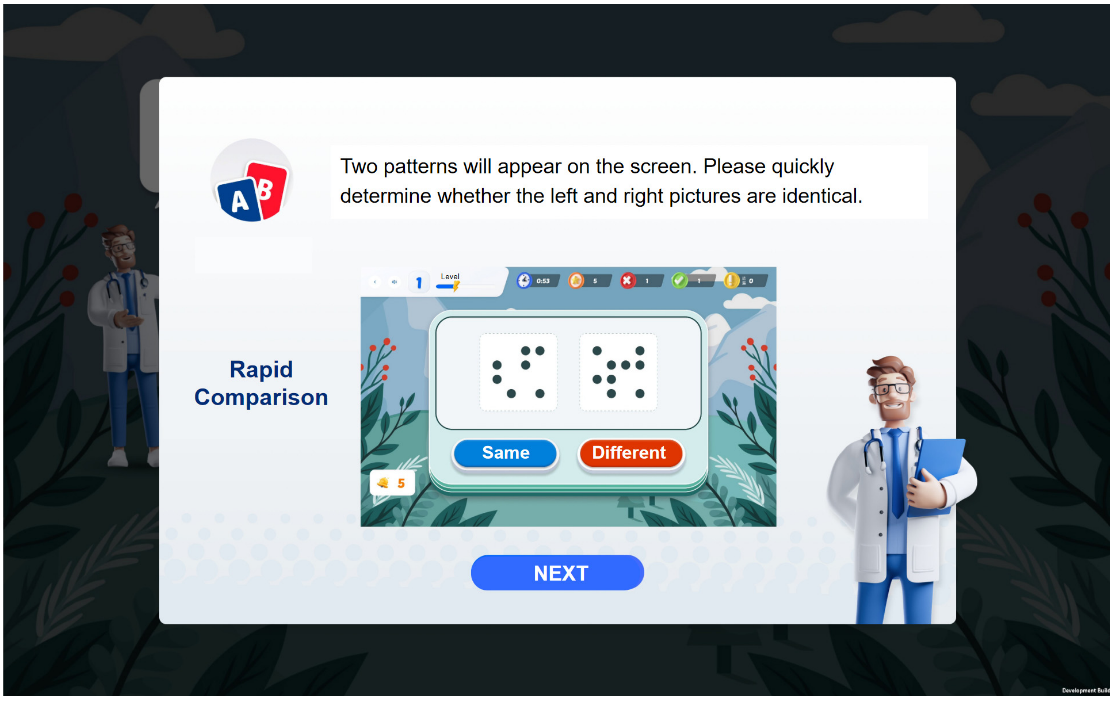

Fine Detail Detection: A series of visually similar insect stimuli are presented sequentially on the screen. Each stimulus appears briefly and disappears before the next one is shown. Some stimuli may be repeated across the sequence.Participants are required to monitor the sequence and identify insects that are identical to previously presented stimuli.

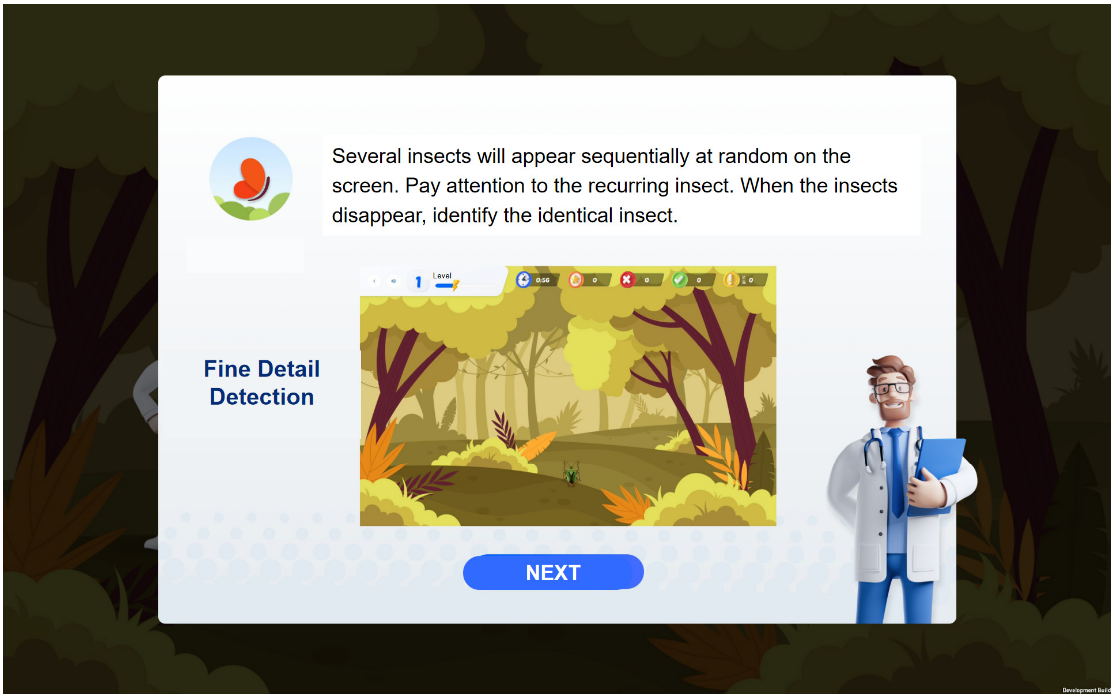

Angles: An angle formed by two lines, along with its degree measure, is presented on the left side of the screen. Participants are required to select a line on the right side that, together with a designated reference line, forms an angle corresponding to the target degree.

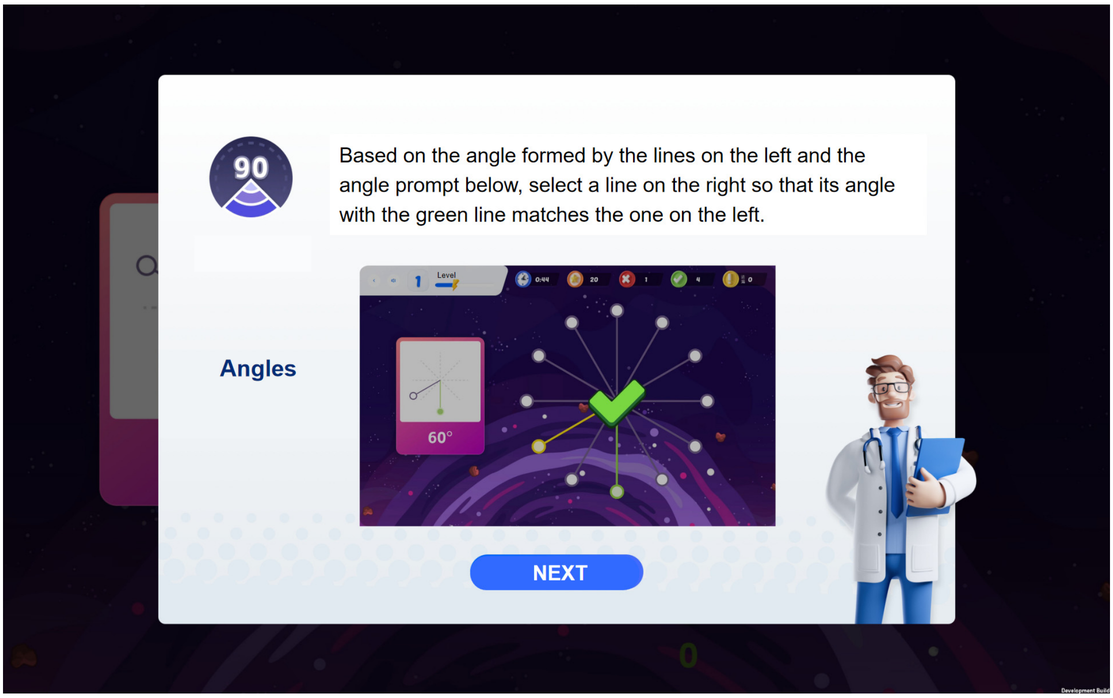

Mystery Garden: A set of insects is randomly displayed on the left side of the screen. Participants are required to drag and position the insects from the right side so that their locations match the corresponding positions of the insects on the left as accurately as possible.

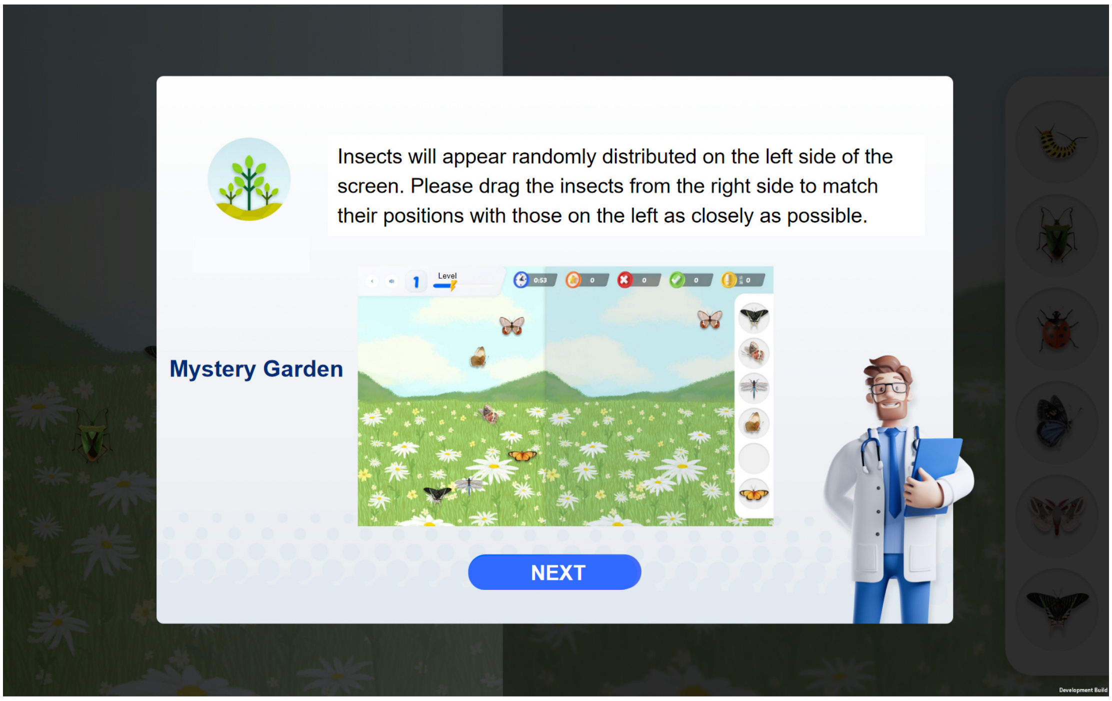

Avian Visual Discrimination: A circular array of bird stimuli is presented on the screen, with each bird displaying a distinct posture. Two of the birds differ from the others in color. After a brief presentation period, the positions of all birds are occluded. Participants are required to identify the spatial locations (regions) where the two deviant birds were originally positioned.

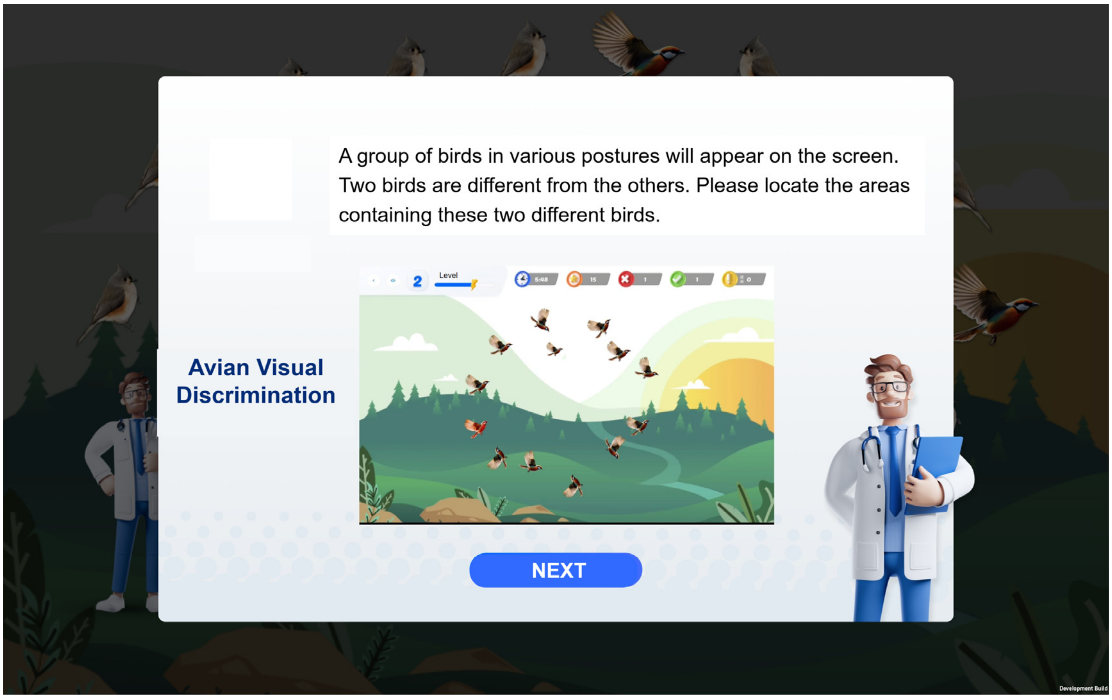

Face-Name Association Task: Three face stimuli are presented sequentially on the screen, each accompanied by a corresponding name displayed below the image. After viewing each face-name pair, participants indicate successful encoding by selecting a “Remembered” button, and then proceed to the next stimulus. Following the encoding phase, a retrieval phase is initiated. Participants are presented with a previously viewed face on the left side of the screen and are required to select the corresponding name from multiple options displayed on the right.

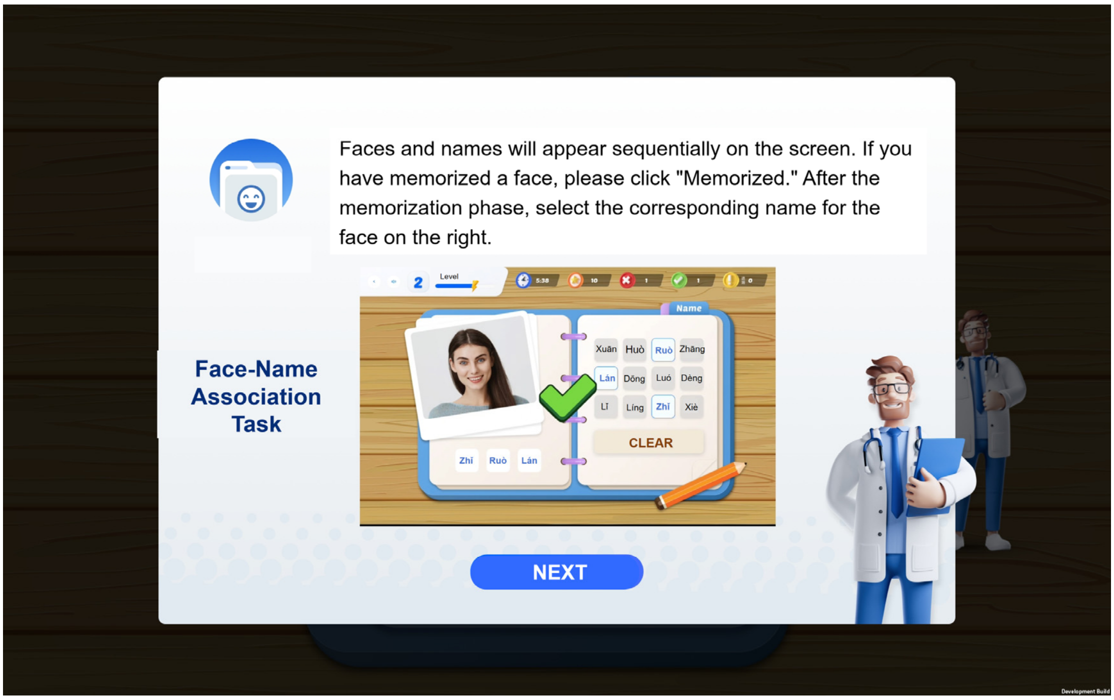

Supplement: Supplementary file 1 [file brainsci-16-01125-s001.zip › brainsci-4601984-supplementary.pdf]
